# Accurate Prediction of Ion Mobility Collision Cross-Section Using Ion’s Polarizability and Molecular Mass with Limited Data

**DOI:** 10.1021/acs.jcim.3c01491

**Published:** 2024-02-23

**Authors:** Pattipong Wisanpitayakorn, Sitanan Sartyoungkul, Alongkorn Kurilung, Yongyut Sirivatanauksorn, Wonnop Visessanguan, Nuankanya Sathirapongsasuti, Sakda Khoomrung

**Affiliations:** † Siriraj Center of Research Excellence in Metabolomics and Systems Biology (SiCORE-MSB), Faculty of Medicine Siriraj Hospital, Mahidol University, Bangkok 10700, Thailand; ‡ Siriraj Metabolomics and Phenomics Center, Faculty of Medicine Siriraj Hospital, Mahidol University, Bangkok 10700, Thailand; § National Center for Genetic Engineering and Biotechnology (BIOTEC), Pathumthani 12120, Thailand; ∥ Section of Translational Medicine, Faculty of Medicine Ramathibodi Hospital, Mahidol University, Bangkok 10400, Thailand; ⊥ Research Network of NANOTEC - MU Ramathibodi on Nanomedicine, Bangkok 12120, Thailand; # Department of Biochemistry, Faculty of Medicine Siriraj Hospital, Mahidol University, Bangkok, 10700, Thailand; ∇ Center of Excellence for Innovation in Chemistry (PERCH−CIC), Faculty of Science Mahidol University, Bangkok 10400, Thailand

## Abstract

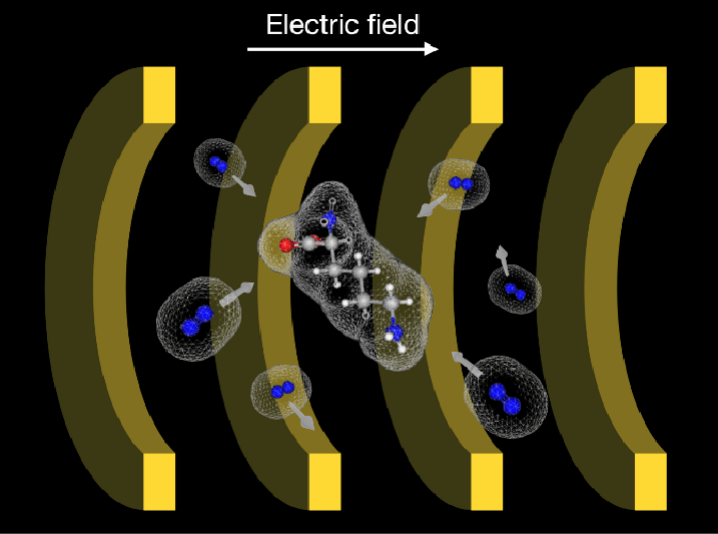

The rotationally averaged collision cross-section (CCS) determined by ion mobility-mass spectrometry (IM-MS) facilitates the identification of various biomolecules. Although machine learning (ML) models have recently emerged as a highly accurate approach for predicting CCS values, they rely on large data sets from various instruments, calibrants, and setups, which can introduce additional errors. In this study, we identified and validated that ion’s polarizability and mass-to-charge ratio (*m*/*z*) have the most significant predictive power for traveling-wave IM CCS values in relation to other physicochemical properties of ions. Constructed solely based on these two physicochemical properties, our CCS prediction approach demonstrated high accuracy (mean relative error of <3.0%) even when trained with limited data (15 CCS values). Given its ability to excel with limited data, our approach harbors immense potential for constructing a precisely predicted CCS database tailored to each distinct experimental setup. A Python script for CCS prediction using our approach is freely available at https://github.com/MSBSiriraj/SVR_CCSPrediction under the GNU General Public License (GPL) version 3.

## Introduction

In recent years, ion-mobility spectrometry (IMS) has been used as an additional mass analyzer to improve the separation and identification performance of mass spectrometry (MS). Upon reaching the ion-mobility (IM) chamber, analytes are subjected to an electric field as they travel through a buffer gas, which leads to their separation. The time taken by the ions to travel across the IM chamber is typically converted to an orthogonal molecular descriptor known as ‘a rotationally averaged collision cross-section’ (CCS).^1,2^ CCS is a physicochemical property that is currently known to depend on the shape and size of the ion.^3^ CCS values, in addition to the retention time, accurate measurement of mass-to-charge ratios (*m*/*z*), and tandem mass spectrometry (MS/MS), have been increasingly used as an additional parameter to increase the confidence in compound identification.^4,5^ However, CCS values have been experimentally determined for only a small fraction of metabolites (∼3,000),^6−8^ which is significantly lower than the total number of metabolites expected in the human body (220,945 metabolites as of March 2023).^9^

To overcome this issue, machine learning (ML)^6,7^ and deep learning (DL)^10^ algorithms and various CCS prediction software^11−14^ were developed. Although ML and DL have demonstrated superior accuracy compared to prediction software that calculates low-field CCS using the trajectory method,^6^ the input parameters used for these models primarily rely on a set of molecular quantum numbers (MQNs),^7^ such as atom counts, bond counts, polarity counts, and topology counts,^15^ which together represent the two-dimensional chemical structure of molecules.^6,7,10^ Because these models are constructed based on a large number (15–50) of input features,^6,7^ they require a large training data set. Thus, these models were trained by using the experimental CCS values obtained from various laboratories, each with its own setup and protocols. Therefore, the models may be subject to additional errors because the CCS value of a molecule may vary depending on the type of IM,^16^ experimental setup,^17^ and CCS calibrants used.^3,18^ Predicting the CCS values of all compounds from a single experiment, although ideal, can be challenging. To construct an accurate prediction model with a limited number of experimental CCS values, it is necessary to identify molecular descriptors that significantly influence CCS values rather than relying on a set of MQNs as inputs for prediction models.

The motion of ions through an IM chamber can be influenced by various factors. First, an ion with a larger mass moves more slowly than an ion with a smaller mass in the same electric field. The movement of ions in an IM chamber primarily depends on their molecular size. A larger ion occupies more space and is more likely to collide with buffer gas molecules in the IM chamber, thereby slowing their movement.^19^ The electric fields in the IM chamber can also induce electric dipoles in the analyte ions and buffer gas molecules. The dipole–dipole interactions between the molecular ions and buffer gas molecules enhance their chances of collision, which slightly alters the CCS values.^20−22^ Two experimental studies^23,24^ previously reported changes in the CCS values when the polarizability of the buffer gas varied. This is because ions with higher polarizabilities have larger dipole moments in the IM chamber and interact more with the polarized buffer gas molecules. In addition, the ion charge state influences the CCS value.^25^

Although it is currently accepted that the CCS values of a molecule depend on its shape,^23,26,27^ no study has quantitatively explored the effect of molecular shape on its CCS. Previous studies have revealed only slight variations in the CCS values across isomeric molecules, such as between betamethasone and dexamethasone^23^ or thyroglobulin and ovalbumin.^27^ The variations in their CCS values suggested that the shape of the molecules affected their CCS, which was inferred from the fact that isomeric molecules have varied forms. However, because these isomeric molecules possessed different three-dimensional structures, the discrepancies in the CCS values observed in these experiments could be attributed to the differences in their sizes or polarizabilities in addition to potential measurement errors.

This study investigated how physicochemical properties of the analyte ions, such as molecular mass, size, shape, and polarizability, affect experimental CCS values. It is important to note that the effect of the ionic charge strength on the CCS value is not within the scope of the current work, as our primary focus is on small molecules, which are mostly detected in either the +1 or −1 charge state. We quantified and verified whether the experimental CCS value varies with molecule’s size and shape. Except for the *m*/*z* value, this is the first time that these correlations have been investigated for a large sample set of small molecules across various chemical classes. Finally, we demonstrated that a highly accurate and robust CCS prediction model can be constructed based on only relevant physicochemical properties.

## Materials and Methods

### Chemicals, Reagents, and Standards

Liquid chromatography–mass spectrometry (LC-MS)-grade acetonitrile (MeCN), methanol (MeOH), and dimethyl sulfoxide (DMSO) were purchased from RCI Labscan (Thailand) and Fisher Chemicals (United States). Formic acid was purchased from Fisher Scientific (Belgium). Ammonium formate was obtained from Thermo Fisher Scientific (United States). Ammonium acetate was purchased from Loba Chemicals (India). Ultrapure water (H_2_O) was obtained by using a Milli-Q water system (Millipore, USA). Analytical grade or higher purity 125 analytical standards from 28 classes of polar metabolites were purchased from Sigma-Aldrich (Germany). They constituted azoles, benzene and substituted derivatives, biotin and derivatives, carboxylic acids and derivatives, cinnamic acids and derivatives, diazines, dihydrofurans, fatty acids, flavin nucleotides, furans, hydroxy acids and derivatives, imidazopyrimidines, indoles and derivatives, keto acids and derivatives, organic sulfonic acids and derivatives, organonitrogen compounds, organooxygen compounds, peptidomimetics, phenol esters, phenols, phenylpropanoic acids, phenol lipids, pteridines and derivatives, purine nucleotides, pyridines and derivatives, pyrimidine nucleosides, quinolines and derivatives, and steroids and derivatives.

Individual standards were prepared as stock solutions in H_2_O, MeOH, or DMSO, based on their solubilities. For CCS determination, working standard solutions were prepared from 20 to 100 μM. TWIMS was calibrated for mass calibration and CCS measurements using a Major Mix IMS/TOF calibration kit (Waters, United States). Leucine-enkephalin (Waters, United States) was prepared at 200 pg/μL in 50:50 (v/v) MeCN/H_2_O with 0.1% formic acid and used as a lock mass reference for accurate mass calibration.

### UPLC-TWIMS-QToF-MS Analysis

To measure the TWIM CCS values, we used the method proposed by Kurilung et al.^28^ In short, the UPLC system employed in this study utilized an ACQUITY BEH HILIC, 2.1×100 mm, 1.7 μm column (Waters, United States). Two distinct chromatographic conditions, namely, acidic and basic, were implemented. Under acidic conditions, mobile phase A consisted of 95:5 (v/v) MeCN/H_2_O, and mobile phase B comprised 50:50 (v/v) MeCN/H_2_O. Both phases were supplemented with 10 mM ammonium formate (pH 3.0) and 0.125% formic acid. Conversely, under basic conditions, mobile phase A was composed of 95:5 (v/v) MeCN/H_2_O, while mobile phase B contained 50:50 (v/v) MeCN/H_2_O. In both phases, 10 mM ammonium acetate (pH 9.0) and 0.04% ammonium hydroxide were included.

The UPLC system was coupled with a Synapt G2-Si HDMS traveling-wave ion-mobility mass spectrometer (Waters, U.S.A.) for CCS measurements. The measurements were conducted in both positive and negative electrospray ionization (ESI) modes. In the ESI+ mode, the following settings were used: a capillary voltage of 2.5 kV, cone voltage of 30 V, source temperature of 100 °C, and desolvation temperature of 200 °C. For the ESI– mode, the settings were as follows: a capillary voltage of 2.0 kV, cone voltage of 40 V, source temperature of 100 °C, and desolvation temperature of 200 °C. In the TWIMS-ESI+ mode, the parameters included a nitrogen flow rate of 90 mL/min, wave velocity of 800 m/s, wave height of 30 V, trap bias of 35 V, and helium bias of 30 V. For the TWIM-ESI– mode, the parameters were a nitrogen flow rate of 90 mL/min, wave velocity of 1,000 m/s, wave height of 30 V, trap bias of 35 V, and helium bias of 30 V. Data were acquired in the *m*/*z* range of 50–1000 Da using the high-definition MSE (HDMSE) mode with a scan time of 0.2 s. The Progenesis QI software (Nonlinear Dynamics, U.K.) was employed for raw data analysis, including the calculation of CCS values.

### Adduct Structure Optimizations and Molecular Property Calculations via Gaussian09 Software and MarvinSketch

To obtain an accurate three-dimensional (3D) structure of each compound adduct, a simplified molecular-input line-entry system (SMILES) was imported into Avogadro software^29^ to generate a three-dimensional structure of the neutral compound. To minimize the structural energy, a quick structural optimization was performed using the universal force field (UFF). Next, the compound geometry was accurately optimized via Gaussian09 (G09) software^30^ and density functional theory (DFT) at the B3LYP/6-31+G(d,p) level of theory. The simulation was performed with a zero-point energy correction, and the keyword ‘pop = (mk, dipole)’ was applied to the Merz–Kollman partial-charge and dipole-moment calculations. The ground-state-optimized geometry of the neutral molecule was confirmed to have no imaginary frequencies. Following the Gaussian simulation, the location for adduct formation was determined by observing the atomic charges of neutral molecules using GaussView software (version 5.0).^31^ To construct [M + H]^+^ and [M + Na]^+^ adducts, additional hydrogen or sodium atoms were added to the neutral structure. In contrast, the most acidic proton was removed from the neutral structure to form a [M – H]^−^ adduct. The charges of the newly formed adducts were identified as +1 for [M + H]^+^ and [M + Na]^+^ and −1 for [M– H]^−^. The adduct geometry was further optimized through G09 using the same theory as that used for the neutral molecules. Finally, the G09 output file was exported to Vega ZZ software,^32^ where the physicochemical properties, such as the VdW volume, VdW surface area, ovality, and radius of gyration of the adduct, were calculated. The molecular volume and surface area were calculated using a probe radius of 0. We used MarvinSketch^33^ to calculate the molecular polarizability of the adduct compounds. Initially, we initiated the chemical structure of each adduct in MarvinSketch software. Subsequently, we utilized the Polarizability Plugin and selected ‘take 3D geometry (Thole)’ to calculate the polarizability. In total, the molecular properties of 197 adducts from three types, [M + H]^+^, [M + Na]^+^, and [M – H]^−^, were calculated.

### Support Vector Regression for CCS Prediction

To predict CCS values based on molecular properties, we employed support vector regression (SVR) provided by the sci-kit learn library.^34^ Calculated molecular properties and experimental CCS data were used for model building and performance evaluation. In particular, we analyzed the entire data set from each ion mode individually.

To evaluate the performance of the model built from each combination of molecular properties, each data set was randomly partitioned into 80% for training and 20% for testing. This training–testing process was repeated 10,000 times to eliminate bias from random sampling. Because the number of data points available for each ion mode was different, we randomly selected 70 data points for training and 18 data points for testing the model for a fair comparison. This is similar to performing an 80/20 train–test split but with the assumption that the positive mode consists of only 88 compounds, which is the same number of compounds as the negative mode. Before training the model for each iteration, we performed a hyperparameter tuning on each of the training data sets using 5-fold cross-validation (CV) and following tuning parameters: ‘kernel:’ [″rbf,” ‘C:’ [100, 1000, 10000, 100000, 1000000],’gamma:’ [0.01, 0.1, 1], and’epsilon:’ [0.01, 0.1, 1]. Min–max scaling was used for feature scaling. The optimized parameters were used to train each SVR prediction model.

For each testing compound, the predicted CCS values and those obtained from AllCCS and CCSbase (obtained in January 2021) were compared with the experimental values. The mean relative error (MRE) represents the prediction performance of the SVR models. All analyses were performed by using the Python programming language.

### Determining Important Variables via Multiple Linear Regression with Interactions

To determine the dependence of the CCS values on *m*/*z* or ovality, we performed multiple linear regression (MLR)^35^ using the generalized linear model (GLM) function in the statsmodels Python package.^36^ For the GLM function, we specified the model family as Gaussian and used the following form:
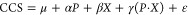
1

where μ is the intercept, *P* is the polarizability, α is the coefficient of polarizability, *X* is *m*/*z* when testing the effect of mass or ovality or the effect of shape, β is the coefficient of *m*/*z* or ovality, γ is the coefficient of interaction, and ε is the error. The MLR analyses in this work were performed on molecules with *m*/*z* in the range of 87.04–385.35, which is the range where most of our compounds were densely populated.

## Results and Discussion

### Experimental CCS Measurement and Quantum Chemistry Calculation

**Figure 1:**
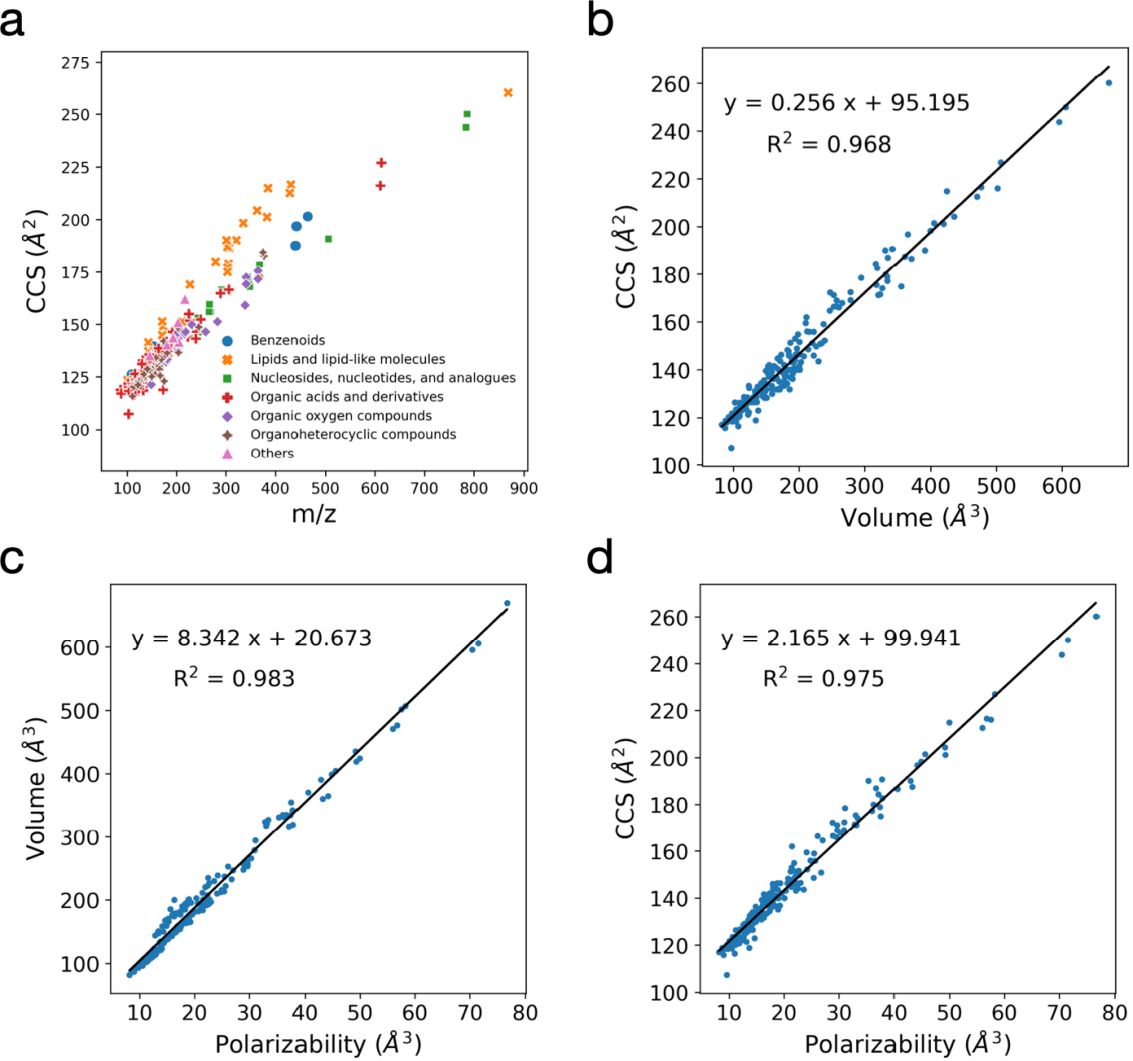
Linear correlations demonstrating dependencies of CCS values of small molecules on their volumes and polarizabilities. (a) Scatter plot between *m*/*z* and CCS of the compounds measured in our TWIM experiment. (b) Linear correlations between our experimentally measured TWIM CCS values and VdW volume. (c) Linear correlations between the VdW volume and polarizability. (d) Linear correlations between the TWIM CCS values and polarizability.

We conducted experimental CCS measurements using ultrahigh-performance liquid chromatography coupled with traveling-wave ion-mobility mass spectrometry (UPLC-TWIM-MS). We measured the CCS values of 125 reference standards from 28 chemical classes in both positive (ESI+) and negative (ESI−) electrospray ionization modes using nitrogen gas (N_2_) as the buffer gas. This resulted in CCS values of 197 adducts (52 from [M + H]^+^, 57 from [M + Na]^+^, and 88 from [M – H]^−^) with *m*/*z* in the range of 87.0437–868.1255 (Figure 1a). The physicochemical properties of these adduct compounds, such as van der Waals (VdW) volumes, VdW surface areas, ovalities, radii of gyration, and polarizabilities, were analyzed using quantum chemistry calculations (Materials and Methods and Supplementary Figure 1). Notably, these molecular properties were calculated in the adduct form because the process of gaining or losing atoms during adduct formation alters their molecular properties. All the measured Traveling-Wave Ion-Mobility (TWIM) CCS values and calculated physicochemical properties are listed in Supplementary Table 1.

### Strong Correlation Between Ion Sizes and Their CCS Values

Because the CCS of a molecule depends on its size,^37^ we observed strong correlations between the CCS values and VdW volume (*R*^2^ = 0.968) (Figure 1b) and VdW surface area (*R*^2^ = 0.964) (Supplementary Figure 2a). As the VdW volumes and surface areas of the adduct molecules also exhibited a very strong linear correlation (*R*^2^ = 0.993) (Supplementary Figure 2b), we were unable to verify whether the VdW volume or surface area was better at predicting the CCS value. Another size-related property of interest is the radius of gyration, which is frequently used to measure the structure compactness.^38^ However, when compared with the VdW volume and surface area, the radii of the gyration molecules greatly underrepresented the CCS values (*R*^2^ = 0.731), as shown in Supplementary Figure 2c. This may be because the radius of gyration represents the mass distribution more than the actual size of the molecule, despite being related to size.

Although polarizability is often used to describe the dipole–dipole interaction, it is substantially correlated with spatial size, as demonstrated in previous studies,^37,39,40^ which showed strong correlations (*R*^2^ = 0.962–0.978) between polarizability and volume. This is because the electron volumes of larger molecules are larger than those of smaller molecules.^41^ Similar to previous studies, the polarizabilities of the ions found in our study were significantly correlated with their volumes (*R*^2^ = 0.983) (Figure 1c), demonstrating that polarizability conveys substantial information regarding the spatial volume of ion molecules. Because the ion polarizability also portrayed its volume, we evaluated this physiochemical property and found that our CCS values were highly dependent on the polarizability, with a linear correlation (*R*^2^ = 0.975) (Figure 1d), consistent with a recent study.^42^ This correlation was stronger than that between the CCS values and VdW volume, which might be because the size and dipole–dipole interaction, the two main parameters influencing the mobility of the IM chamber, are both covered by polarizability.

### Polarizability Is the Most Accurate Predictor of CCS Value

**Figure 2:**
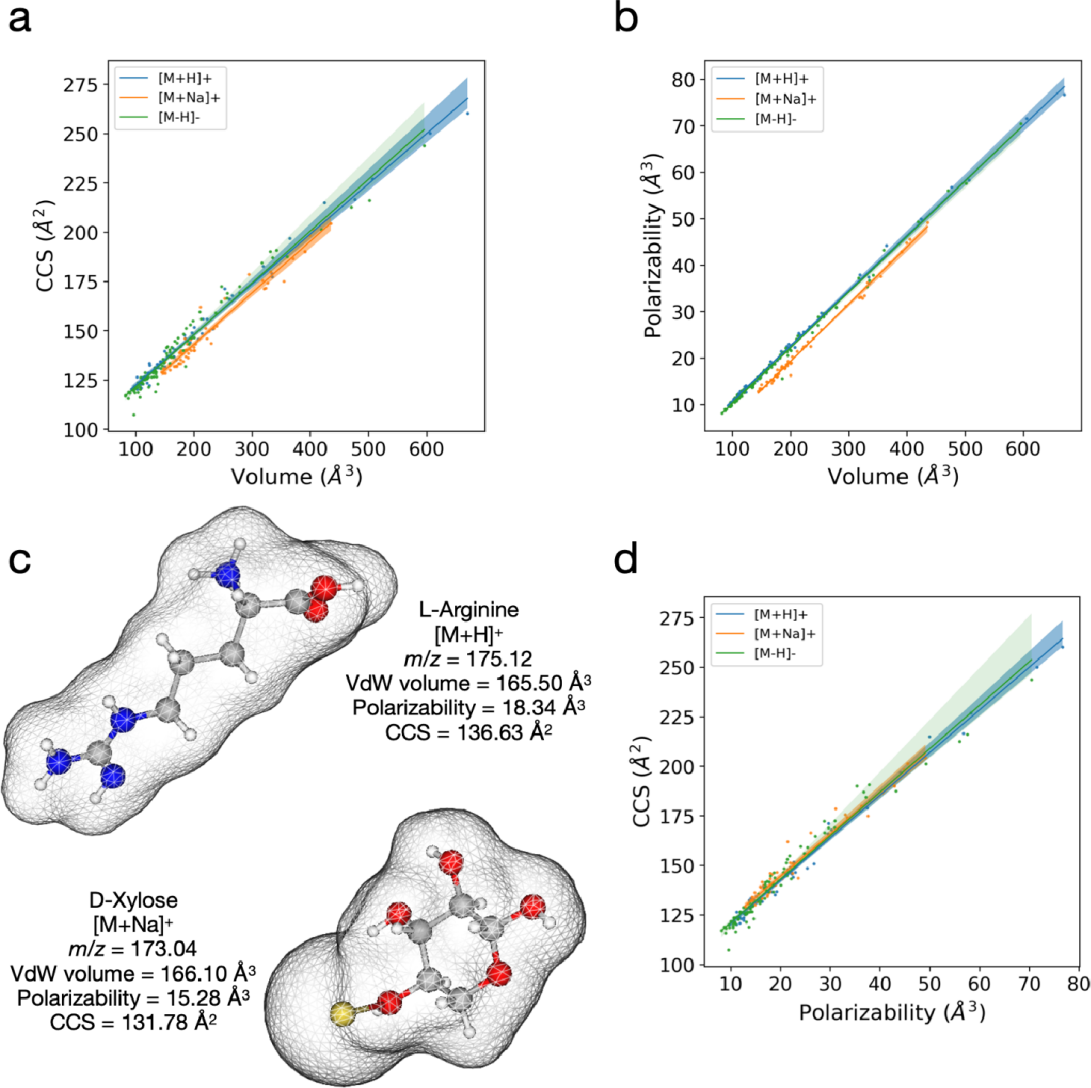
CCS value of a small molecule depends on its polarizability over its volume. (a,b) Separate linear regressions between (a) volume vs CCS values and (b) volume vs polarizability were performed on data for each adduct type. (c) Illustration of the molecular geometries for protonated l-arginine and sodium adduct d-xylose. Although they are similar in volumes, sodium adduct d-xylose possesses a smaller polarizability and CCS value than the protonated l-arginine. (d) Linear regressions between polarizabilities and CCS values for each adduct type. Shaded areas represent 99% confidence intervals. Data set was constructed from 197 adducts obtained from our TWIM experiment.

We postulated that the polarizability of a small molecule may be a better predictor of its CCS value than the VdW volume, because the polarizability of the ion contains information on both its size and electric dipole strength. To investigate this, we divided our data into three groups of adducts ([M + H]^+^, [M + Na]^+^, and [M – H]^−^) and reexamined the linear relationships. We observed a clear downward shift in the correlation between the volumes and CCS values in the sodium adducts, relative to that in the other two adduct types (Figure 2a). Based on this shift, the sodium adduct molecule has a lower CCS value than a protonated or deprotonated adduct of an equal volume. The lower CCS values of the sodium adducts can be explained by evaluating the linear correlations between polarizability and volume, where the correlation for the sodium adducts shifts downward in relation to those for the other two types of adducts (Figure 2b). As polarizability contains information on both size and dipole–dipole interactions, this downward shift in Figure 2b indicates that the sodium adduct has a slightly weaker dipole–dipole interaction with gas molecules (owing to its lower polarizability) than a different protonated or deprotonated adduct of the same volume. The sodium adduct molecule interacts less with the N_2_ gas because of weaker dipole–dipole interactions than the protonated or deprotonated adducts of the same volume, resulting in a smaller drift time and a lower CCS value (the shift in Figure 2a). For example, as shown in Figure 2c, the sodium-adduct d-xylose exhibits a lower CCS value than the protonated l-arginine (131.78 Å^3^ vs 136.63 Å^3^) because the sodium-adduct d-xylose have a smaller polarizability than the protonated l-arginine (15.28 Å^3^ vs 18.34 Å^3^) despite being comparable in volumes (166.10 Å^3^ vs 165.50 Å^3^). It is important to note that the difference in CCS between the sodium-adduct d-xylose and protonated l-arginine is not due to the slight difference in their *m*/*z* values. This is based on a comprehensive analysis of the effect of mass on CCS values, which will be provided in the next section.

In contrast to the volume, the linear correlations between the CCS values and polarizabilities of all three adduct types were almost equal and well within the 99% confidence interval (Figure 2d). Thus, we conclude that the CCS value of a small molecule is more closely related to its polarizability than its volume. This is because the ion polarizability contains information on its VdW interactions (size) and the small contribution from the dipole–dipole interactions, which is supported by a number of earlier computational studies^20−22^ where compound interactions were modeled as the sum of the short-range VdW potential and the long-range ion-induced dipole potential.

### Traveling-Wave Ion-Mobility CCS Value Independent of Shape but Not Mass After Accounting for Polarizability

**Figure 3:**
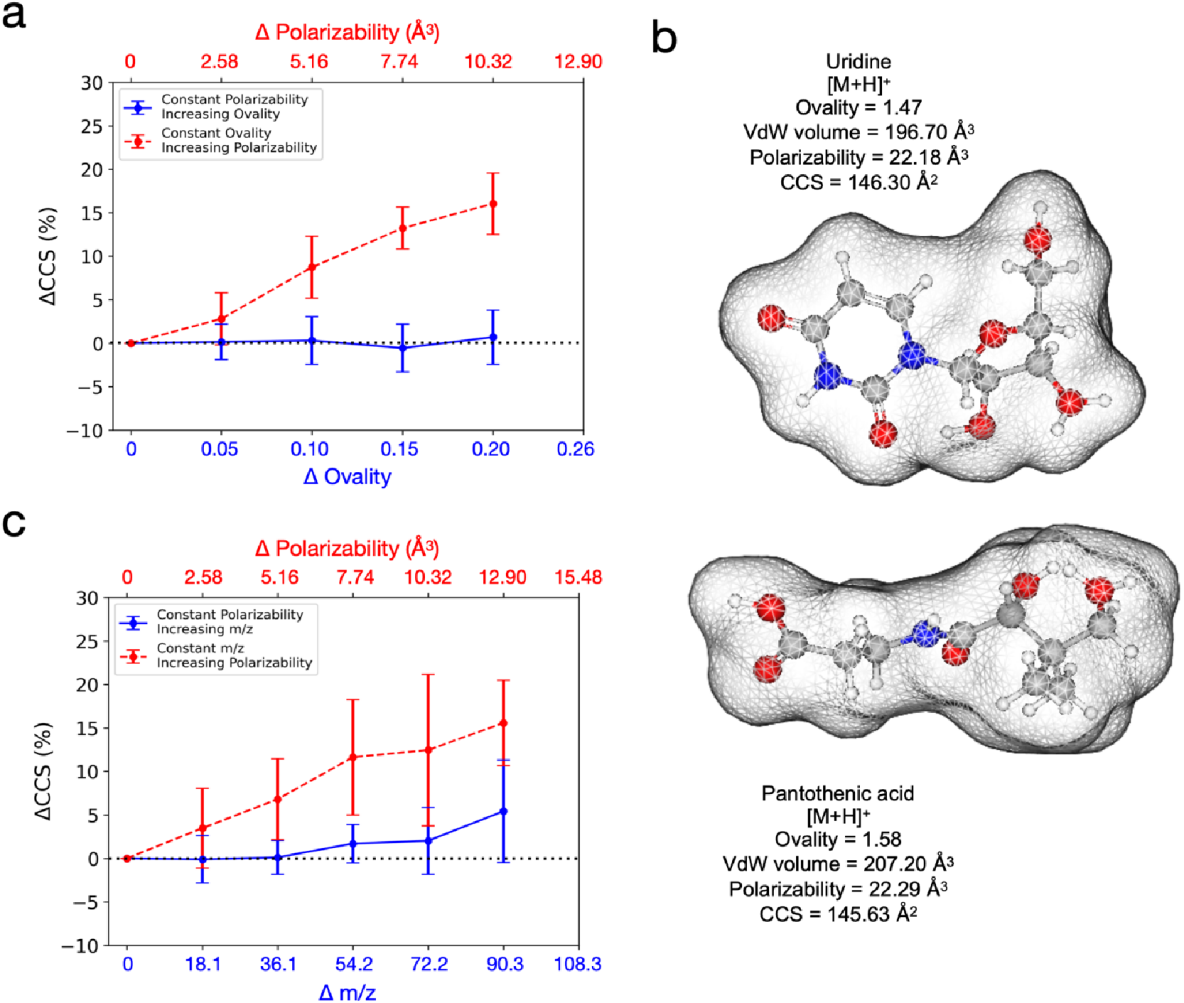
Effect of shape and mass of a small molecule on its experimentally measured TWIM CCS values. (a) Heat map analysis showing changes in CCS values as polarizability increases and ovality stays constant (red-dashed line), and as ovality increases and polarizability stays constant (blue-solid line). (b) Illustration of molecular geometries of protonated uridine and protonated pantothenic acid, which possess different shapes but are similar in polarizabilities and CCS values. (c) Heat map analysis showing changes in CCS values as polarizability increases and *m*/*z* stays constant (red-dashed line), and as *m*/*z* increases and polarizability stays constant (blue-solid line). Plots were constructed based on 197 adducts obtained from our TWIM experiment.

As we determined that polarizability is the strongest predictor for CCS values, our next step is to explore other physicochemical properties that can offer the Supporting Information to polarizability. First, we explored the effect of shape on the CCS value after accounting for polarizability. To study the effect of the shape of a molecule on its CCS value, we quantified the shape as its ovality, which denotes the degree to which the shape of an object deviates from that of a sphere.^43^Supplementary Figure 3 shows geometrical structures of molecules with various ovalities. By observing the heat map of the CCS values between the ovalities and polarizabilities (Supplementary Figure 4), we were able to compare the CCS values of the molecules with different ovalities (shapes) but similar polarizabilities (similar sizes and dipole–dipole interactions). If the shape of an ion provides an extra contribution to its CCS in addition to its polarizability, we hypothesized that molecules with similar polarizabilities but different ovalities would have distinct CCS values. As shown in Figure 3a, we performed an analysis on the heat map data as described in Supporting Information Section 1 to determine the change in the CCS values. As expected, the CCS value for molecules with similar ovalities increased as the polarizability increased (red dashed line, Figure 3a). However, we found no significant difference in the CCS values for molecules with comparable polarizabilities as the ovality increased (blue solid line, Figure 3a). This result strongly indicates that the CCS values of two small molecules with different geometries are comparable if their polarizabilities are similar. As shown in Figure 3b, protonated uridine and protonated pantothenic acid have vastly different shapes but similar CCS values, because of their similar polarizabilities. In support of this, we conducted a MLR analysis^35^ and verified that polarizability was a significant predictor of CCS (*p* = 0.000), whereas ovality was not (*p* = 0.534). Based on the presented results, we inferred that the shape of a small molecule has no major effect on its TWIM CCS.

Next, we investigated the effect of mass on the CCS values of small molecules without the influence of their polarizabilities. As all our ions were in either the +1 or −1 charge state (*z* = 1), *m*/*z* essentially corresponded to the ion masses. We created a heat map of the CCS values between *m*/*z* and polarizability (Supplementary Figure S5) and analyzed it, as shown in Figure 3c. We discovered that the CCS values of the ions with comparable *m*/*z* values increased as the polarizability increased. In contrast to the ovality, we also observed a slight increase in the CCS values as *m*/*z* increased for molecules with similar polarizabilities. These results were also supported by our MLR analysis of polarizability and *m*/*z*, which demonstrated that both polarizability (*p* = 0.000) and *m*/*z* (*p* = 0.000) were statistically significant predictors of CCS. While the findings in this section suggest that the mass of the molecule does influence the TWIM CCS, additional research involving a wider range of compounds with varying CCS values and masses is required to gain a comprehensive understanding of the interplay between these factors

### Drift-Tube Ion-Mobility (DTIM) CCS Value Independent of Mass and Shape After Accounting for Polarizability

**Figure 4:**
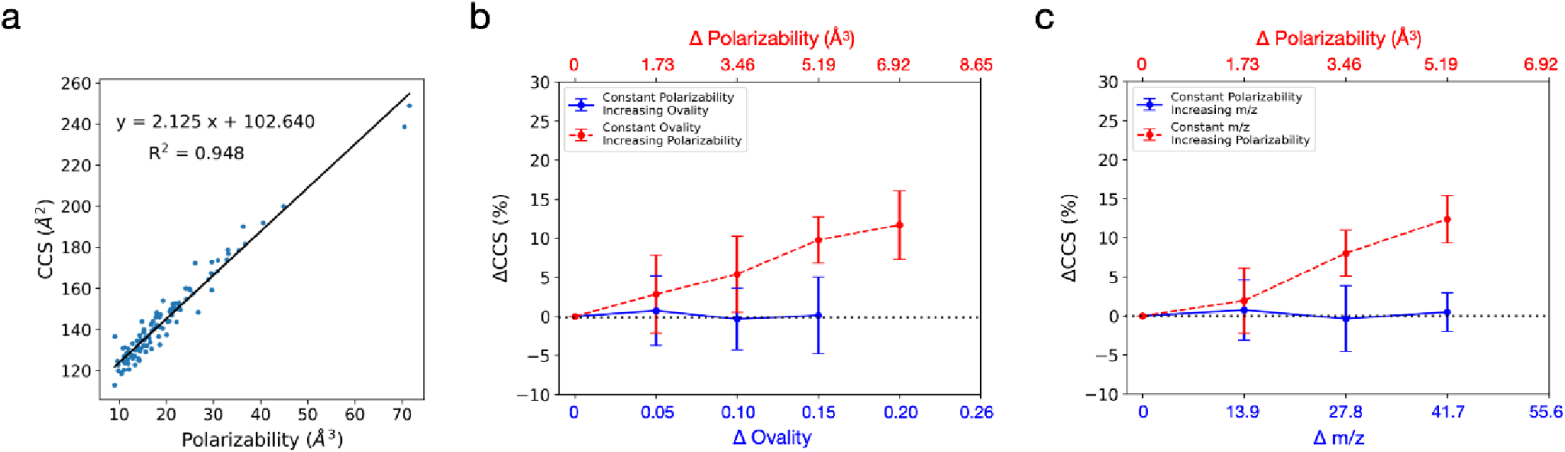
Effect of shape and mass of a small molecule on its DTIM CCS values. (a) Linear correlations between DTIM CCS values and polarizability. (b) Heat map analysis showing changes in CCS values as polarizability increases and ovality stays constant (red-dashed line), and as ovality increases and polarizability stays constant (blue-solid line). (c) Heat map analysis showing changes in CCS values as polarizability increases and *m*/*z* stays constant (red-dashed line), and as *m*/*z* increases and polarizability stays constant (blue-solid line). Plots were constructed based on DTIM CCS values of 108 adduct compounds obtained from CCSbase online database.

In the previous sections, we found that TWIM CCS values depend on polarizability and *m*/*z* but not on ovality. Here, we investigated whether similar behaviors also extend to the drift tube ion mobility (DTIM) instrument, which utilizes a constant electric field instead of a traveling-wave electric field.^16^ We used the experimental DTIM CCS values from the CCSbase database^7^ for the identical adducts investigated in our TWIM experiment. When multiple DTIM CCS values for the same adduct were reported on CCSbase, the average CCS value was used. We obtained DTIM CCS values for a total of 108 adducts (31 [M + H]^+^, 27 [M + Na]^+^, and 50 [M – H]^−^) with *m*/*z* in the range of 103.0393–786.1645. Supplementary Table 2 lists all of the DTIM CCS results and the computed physicochemical parameters relevant to this section. In agreement with our TWIM results, the DTIM CCS values demonstrated a strong linear correlation with polarizability (*R*^2^ = 0.948) (Figure 4a), confirming the importance of polarizability in CCS values.

Next, we analyzed the influence of shape and polarizability on the DTIM CCS values using a heat map (Supplementary Figure 6a). In agreement with the TWIM results, the heat map analysis (Figure 4b) showed that the DTIM CCS values are independent of the shape (ovality) of the molecule. To support this finding, MLR analysis revealed that polarizability (*p* = 0.000) was a statistically significant predictor of CCS, while ovality (*p* = 0.120) was not.

Subsequently, we investigated the effect of mass on the DTIM CCS values without the influence of polarizability using a heat map (Supplementary Figure 6b). Theoretically, the effect of mass on the CCS value should already be corrected by the Mason–Schamp equation,^2^ which is used to calculate the DTIM CCS from the drift time. As expected, the heat map analysis revealed no dependence of the DTIM CCS values on *m*/*z* (Figure 4c). The MLR analysis also indicated that polarizability (*p* = 0.000) was a statistically significant predictor of CCS values, and *m*/*z* (*p* = 0.263) was not. Although this mass correction by the Mason–Schamp equation should be transferred to the TWIM CCS values when constructing the TWIM CCS calibration curve,^1^ the traveling-wave electric field also introduces additional separations based on ion species and traveling-wave settings.^44^ It is possible that the minor mass effect on the TWIM CCS values shown in Figure 3c was a result of the divergence of the TWIM CCS values from their DTIM values (2.34% mean relative error (MRE)) (Supplementary Figure 7a) owing to the additional ion separation in the TWIM chamber. This deviation in CCS values between DTIM and TWIM has been reported in several studies.^16,45^

### Accurate CCS Prediction by Polarizability and *m*/*z* Parameters

By examining the experimental data accessible on CCSbase,^7^ we found that experimental CCS values from different laboratories and experimental setups vary substantially (Supplementary Figure 7b), as also mentioned by previous studies.^3,16−18,45^ Thus, it is beneficial for each laboratory to construct an in-house database of predicted CCS values to achieve the most accurate compound identification. To build an accurate model for CCS prediction based on a limited number of reference standards available in-house, a prediction model must be built using the relevant input parameters.

**Figure 5:**
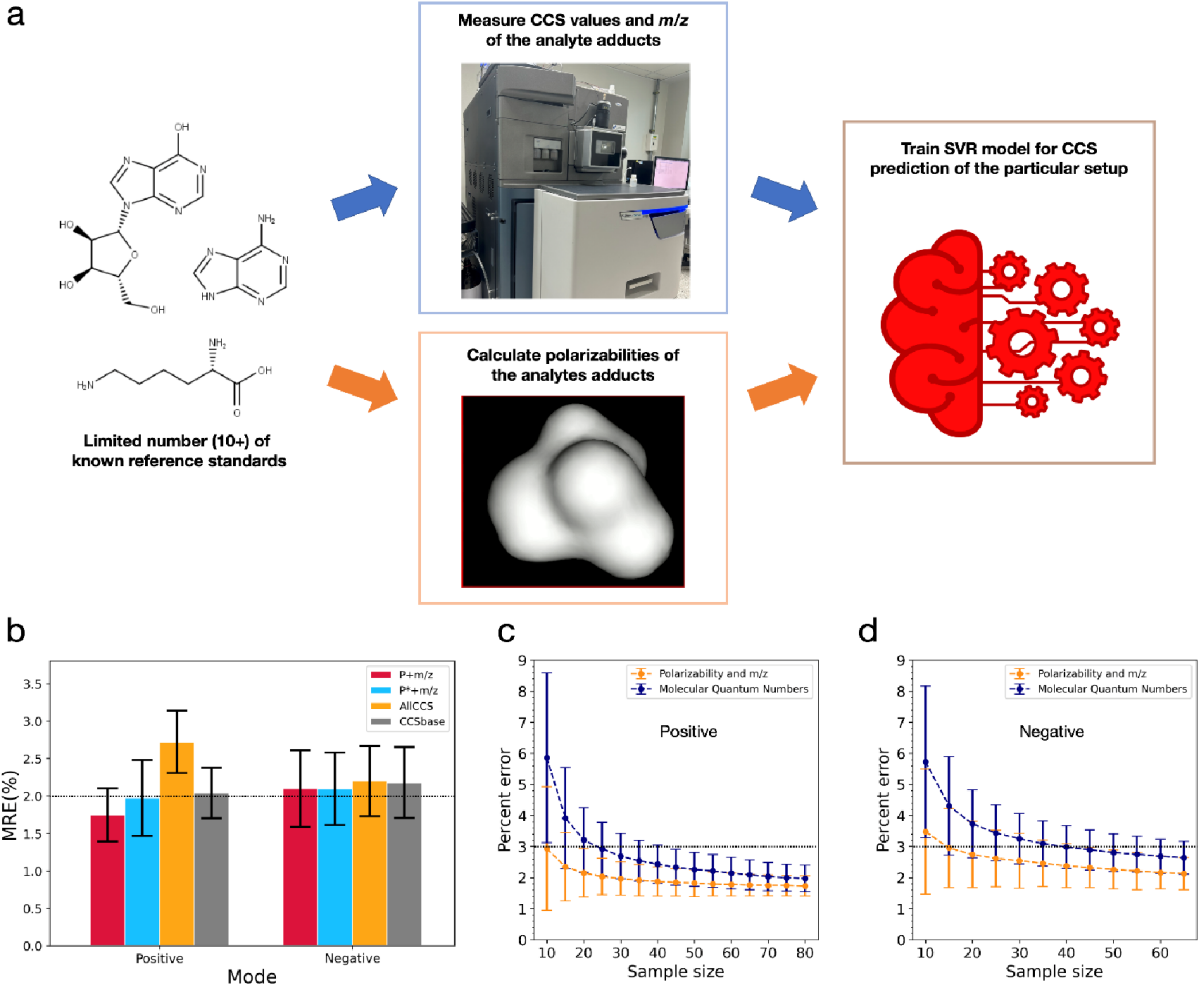
Accurate TWIM CCS prediction of a small molecule based on its polarizability and *m*/*z*. (a) Illustration of simple and accurate support vector regression (SVR) model for CCS prediction using the polarizabilities and *m*/*z* of the analyte adducts. (b) Performance comparison between our approaches, AllCCS, and CCSbase. In the positive ion mode, our SVR model constructed using only the polarizabilities (*P*) and *m*/*z* of the adducts outperforms AllCCS, CCSbase, and the model constructed using the neutral molecules’ polarizabilities (P*) and *m*/*z*. The performances among all the models are comparable in the negative ion mode. (c, d) Accuracy comparisons between the model using adducts’ polarizabilities and *m*/*z* and the model using *m*/*z* and MQNs as inputs for various sizes of training data in the positive (c) and negative (d) ion modes. The number of testing data points was kept constant at 20 for all sample sizes for a fair comparison. The horizontal black line indicates 3.0% MRE.

Based on our findings in the previous sections, we here demonstrate that an accurate and robust CCS prediction model can be constructed using only two meaningful physicochemical properties: adducts’ polarizabilities and *m*/*z*. Figure 5a outlines the simple workflow for constructing this model. Using the CCS values from our TWIM experiment, we validated the performance of our models against two widely used ML CCS prediction tools: AllCCS^6^ and CCSbase.^7^ The full workflow for model construction and performance comparisons is described in Materials and Methods and illustrated in Supplementary Figure 8. As a result, our model, which utilizes only two input parameters, demonstrates high prediction accuracy with an MRE of 1.7 ± 0.3% and 2.0 ± 0.5% in the positive and negative ion modes, respectively. We also observed that the model using the polarizability of the analyte in its adduct form exhibits superior accuracy in positive ion mode compared to the model using the polarizability of the neutral form (Figure 5b). Furthermore, our model outperforms both AllCCS and CCSbase in the positive ion mode and is comparable to them in the negative ion mode (Figure 5b and Supplementary Table 3). However, it is important to note that our model was trained exclusively on data from our instrument, unlike AllCCS and CCSbase, which utilized data from multiple platforms.

Since we focused on only a few crucial input parameters, our approach requires less than 15 experimental CCS values for model training to achieve a 3.0% MRE in both the positive mode (Figure 5c) and negative mode (Figure 5d), significantly fewer than the numbers of CCS values required by the prediction model based on *m*/*z* and molecular quantum numbers (MQNs) as inputs. As our approach is simple and accurate and requires only a small number of experimentally measured CCS values, it demonstrates the potential for constructing an accurately predicted CCS database suitable for each experimental setup, where the number of measured CCS values is typically limited. To utilize our approach in an experiment, one needs to calculate the polarizabilities of ∼20 known molecules, as well as experimentally measure their *m*/*z* and CCS values. Then, an SVR model can be trained for a CCS prediction specific to that experimental condition. Our CCS prediction Python script, based on our approach, is freely available at https://github.com/MSBSiriraj/SVR_CCSPrediction under the GNU General Public License (GPL) version 3.

While our prediction model is slightly more accurate than existing ML-based models, its accuracy can still be improved in several ways. One approach is to identify additional factors influencing CCS beyond polarizability and mass. Additionally, the adducts sometimes may not form at the highest electron density sites. Incorporating the probability of each adduct conformation into the calculation might also improve the accuracy. Moreover, we utilized the physicochemical properties of optimized gas-phase structures without external influences, but it is possible that ionic compounds undergo conformational changes within the IM chamber due to heat and the electric field. Understanding these changes could provide deeper insights into the CCS values. Moreover, certain molecules may exhibit multiple adduct formation sites, potentially affecting their properties. Further studies are needed to explore these factors. Lastly, this study focuses on small molecules, which warrants future investigations into larger organic molecules.

## Conclusion

In this study, we discovered and validated that the CCS value of a small molecule predominantly depends on its polarizability, which reflects its size and dipole–dipole interactions with the buffer gas. We also observed that the shape of a small molecule does not significantly influence the experimental CCS value. The impact of molecular mass on the CCS value, after accounting for polarizability, was observed only in the TWIM experiment and not in the DTIM experiment. This difference may be attributed to the additional ion separation caused by the traveling waves. Furthermore, we demonstrated the capability to construct an accurate CCS prediction model using polarizabilities and *m*/*z*, particularly effective with a limited number of CCS values for training. Our approach highlights the potential for developing a precisely predicted CCS database customized for each experimental setup.

## Data Availability

The python code for CCS prediction using adducts’ polarizabilities and *m*/*z*, instruction manual, and example input files are freely available at https://github.com/MSBSiriraj/SVR_CCSPrediction. Additional relevant information is also available in the Supporting Information.

## Supporting Information Available

Detailed information on our heat map analysis, supplementary figures, and tables containing all the information used in our analyses, SVR model construction, and SVR model performance assessment (our experimentally measured TWIM CCS values, DTIM CCS values obtained from CCSbase, and the quantum chemistry calculated physicochemical properties of the associated adducts) (PDF)
