# Supplementary material for: Accurate Prediction of Ion Mobility Collision Cross-Section Using Ion’s Polarizability and Molecular Mass with Limited Data
Source: J Chem Inf Model. 2024 Feb 23;64(5):1533–42. doi: 10.1021/acs.jcim.3c01491 (PMC10934814; doi:10.1021/acs.jcim.3c01491)
Supplement: Supplementary file 1 — ci3c01491_si_001.pdf [file ci3c01491_si_001.pdf]

Supplementary Information for:

Accurate Prediction of Ion Mobility Collision Cross Section using Ion's Polarizability and Molecular Mass with Limited Data

Pattipong Wisanpitayakorn[1,2], Sitanan Sartyoungkul[1,2], Alongkorn Kurilung[1,2], Yongyut Sirivatanauksorn[1,2], Wonnop Visessanguan[3], Nuankanya Sathirapongsasuti[4,5], Sakda Khoomrung[1,2,6,7,*]

[1]Siriraj Center of Research Excellence in Metabolomics and Systems Biology (SiCORE-MSB), Faculty of Medicine Siriraj Hospital, Mahidol University, Bangkok 10700, Thailand

[2]Siriraj Metabolomics and Phenomics Center, Faculty of Medicine Siriraj Hospital, Mahidol University, Bangkok 10700, Thailand

[3]National Center for Genetic Engineering and Biotechnology (BIOTEC), Pathumthani 12120, Thailand

[4]Section of Translational Medicine, Faculty of Medicine Ramathibodi Hospital, Mahidol University, Bangkok 10400, Thailand

[5]Research Network of NANOTEC - MU Ramathibodi on Nanomedicine, Bangkok 12120, Thailand

[6]Department of Biochemistry, Faculty of Medicine Siriraj Hospital Mahidol University, Bangkok 10700, Thailand

[7]Center of Excellence for Innovation in Chemistry (PERCH-CIC), Faculty of Science Mahidol University, Bangkok 10400, Thailand

Sakda Khoomrung: https://orcid.org/0000-0001-9461-8597

*Correspondence: sakda.kho@mahidol.edu

Supplementary Section 1: Heat map analysis of CCS values between two physio-chemical properties

To better understand the CCS value heat map, we conducted an analysis where we monitored the percent change in CCS values as one parameter remained constant while the other increased. As an example, we used the heat map of CCS values over a range of ovalities and polarizabilities (**Supplementary Figure 4a**). The workflow for this analysis is illustrated in **Supplementary Figure 10**. Each box of the heat map represents the average CCS value of multiple compounds with ovalities and polarizabilities within that bin. To compare whether the CCS values differed for constant polarizabilities but different ovalities, we calculated the percent difference in CCS values between the first box and the other boxes on each row (moving horizontally to the right). For instance, in **Supplementary Figure 4a**, moving one box to the right meant that the ovality increased by 0.05 while the polarizability remained constant. We then averaged the calculated percent differences across all the rows.

To compare CCS values for compounds with similar ovalities but different polarizabilities, we compared the CCS values between the first box of each column and the other boxes on the same column (moving up vertically). For example, in Supplementary Figure 4a, moving one box up meant that the polarizability increased by 2.58 Å^3 while the ovality remained constant. We then calculated the average ± standard deviation values ($n \geq 3$) of the percent differences and plotted them in **Figure 4a**.

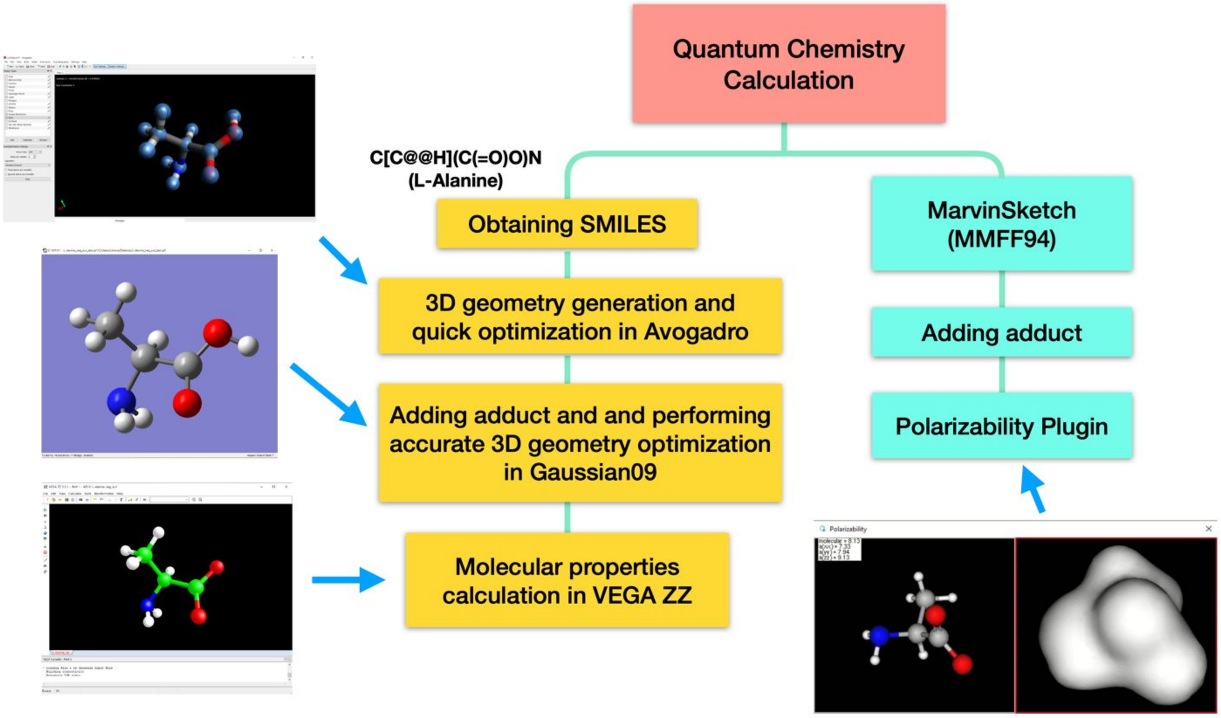

Supplementary Figure 1: Flowchart illustrates molecular property calculation process via Gaussian09 and MarvinSketch software. The process is also assisted by three other software: Avogadro, GaussView5.0, and VEGA ZZ.

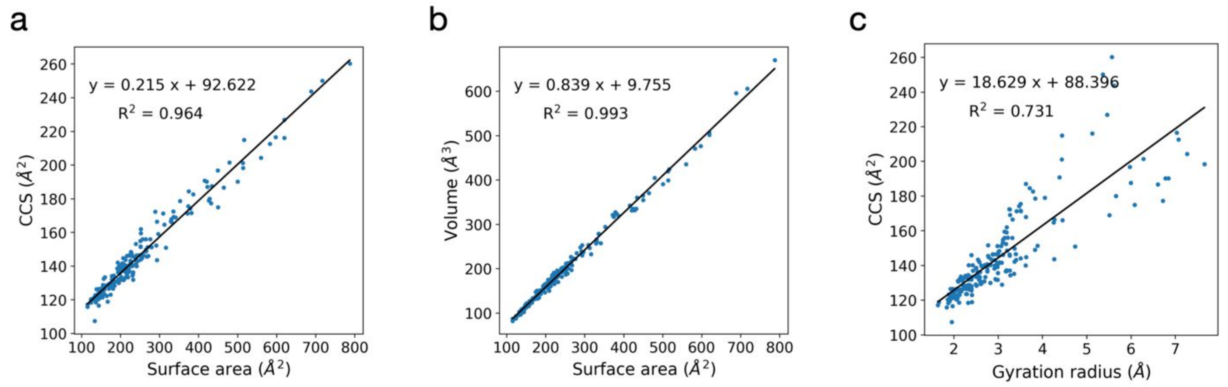

Supplementary Figure 2: Linear correlations used to support our analysis. (a) Linear correlations between the CCS values and VdW surface areas. (b) Linear correlations between the VdW volumes and VdW surface areas. (c) Linear correlations between the CCS values and gyration radius. The plots were constructed based on 197 adduct compounds from our TWIMS experiment.

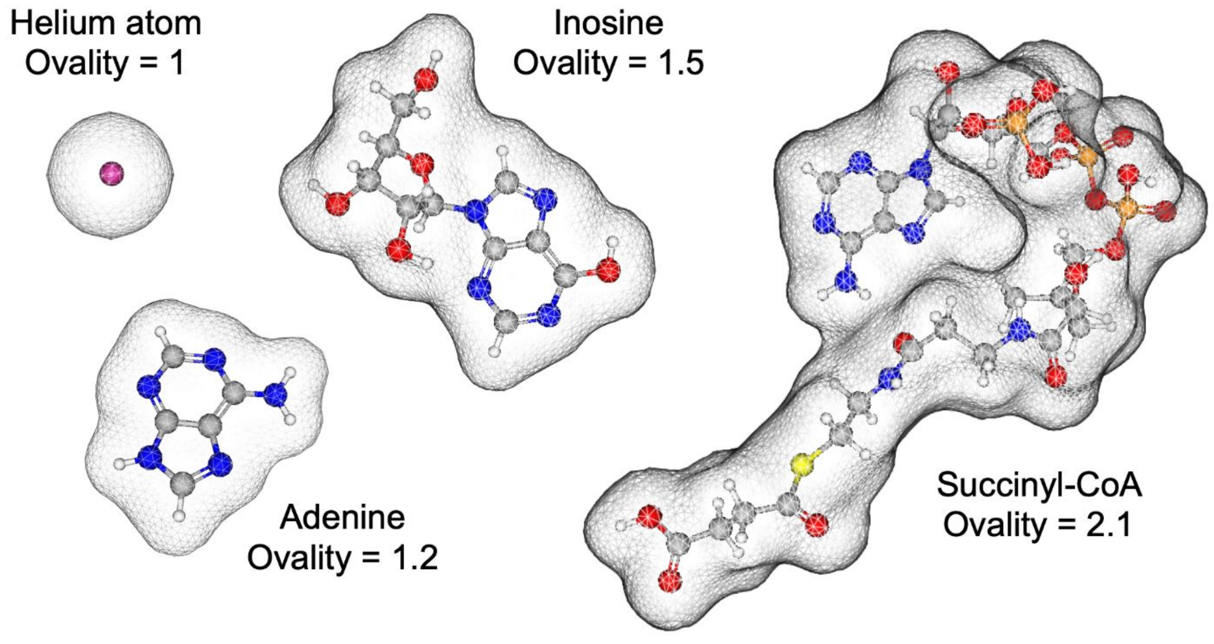

Supplementary Figure 3: Illustration of molecules with various ovalities.

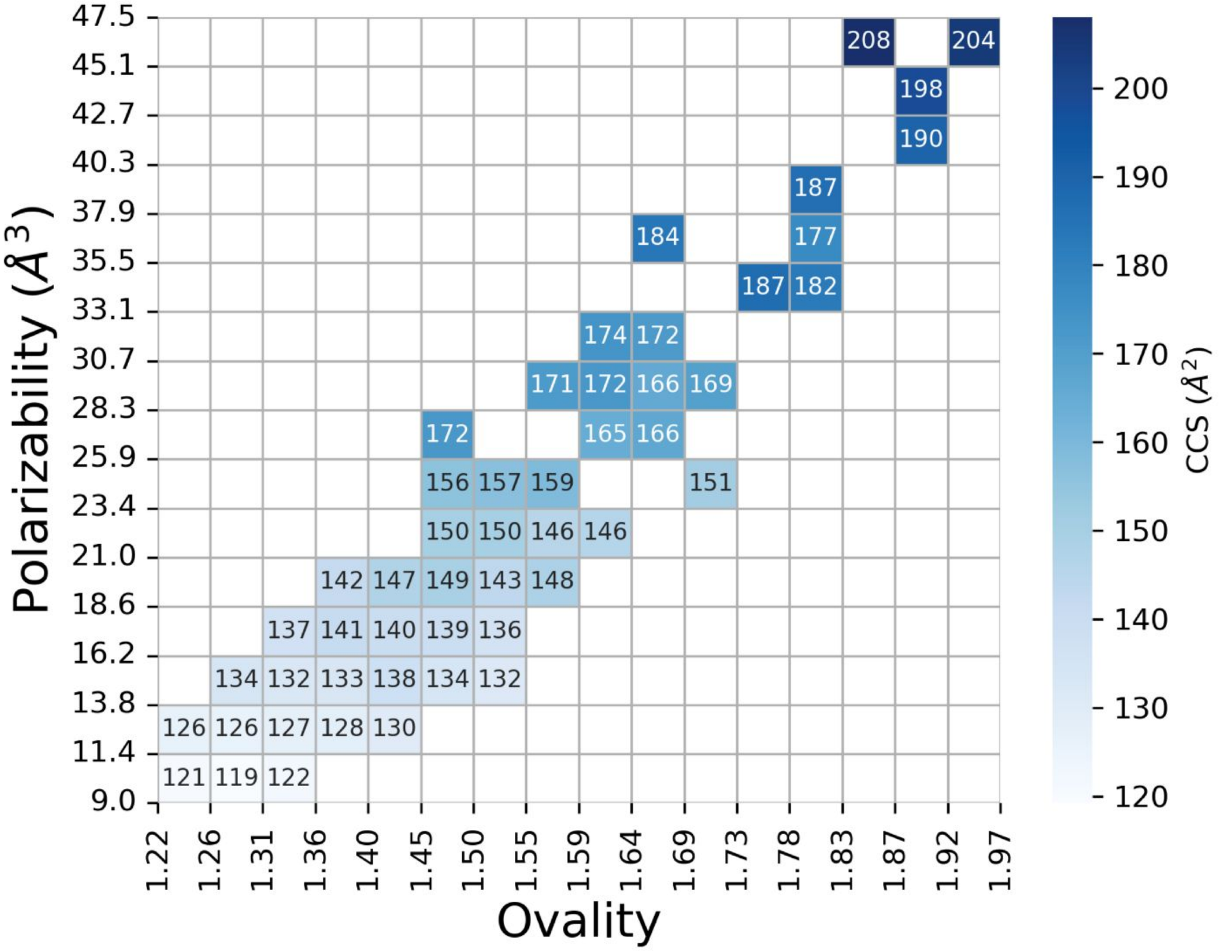

Supplementary Figure 4: Heat map of the CCS values generated over a range of ovalities and polarizabilities.

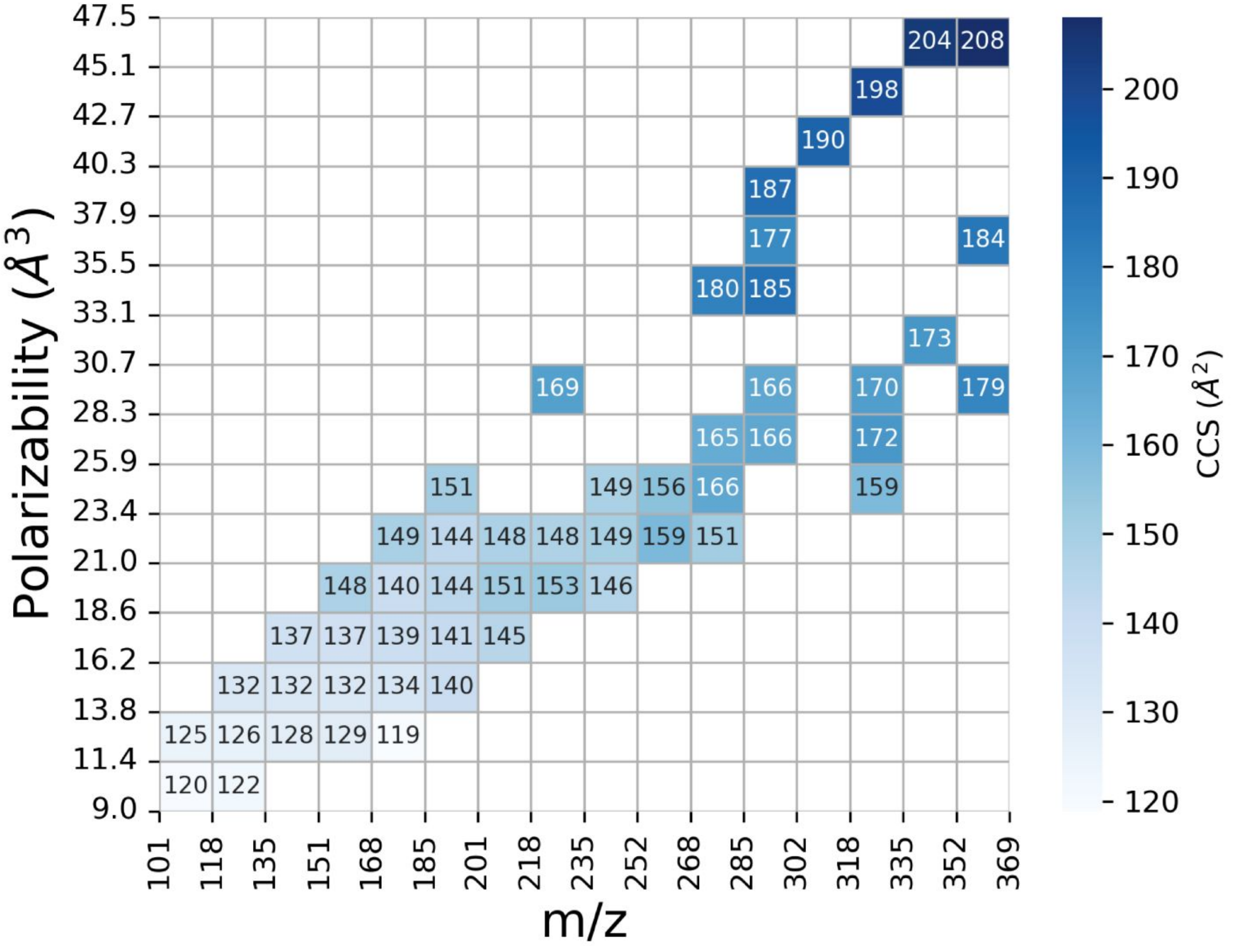

Supplementary Figure 5: Heat map of the CCS values generated over a range of *m/z* and polarizabilities.

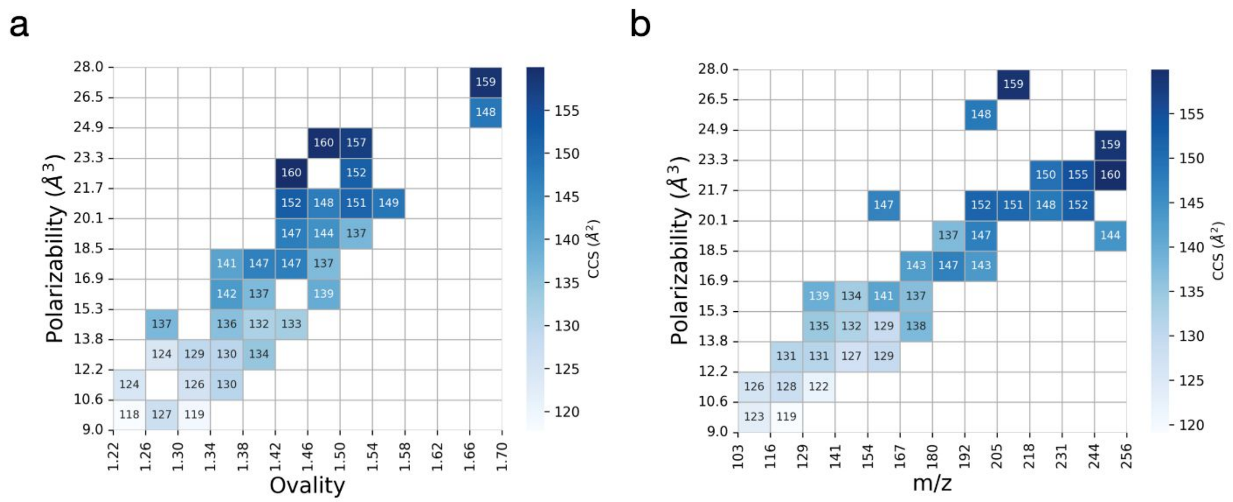

Supplementary Figure 6: Heat maps showing no independences of drift-tube ion mobility (DTIM) CCS values on the ion's shapes and masses. (a) Heat map of the DTIM CCS values generated over a range of ovalities and polarizabilities. (b) Heat map of the DTIM CCS values generated over a range of *m/z* and polarizabilities.

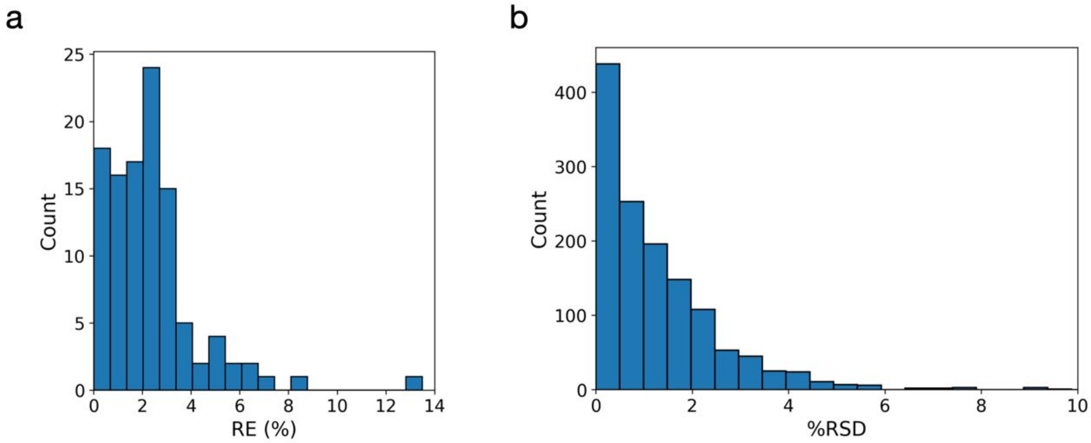

Supplementary Figure 7: Histograms used to support our analysis (a) Deviation in our experimentally measured TWIM CCS values from their DTIM values available on the CCSbase. (b) Deviation in experimental CCS values across different experiments. Histogram shows the percent relative standard deviations (%RSD) of CCS values of the same adduct compounds reported by two or more experiments on the CCSbase experimental database.

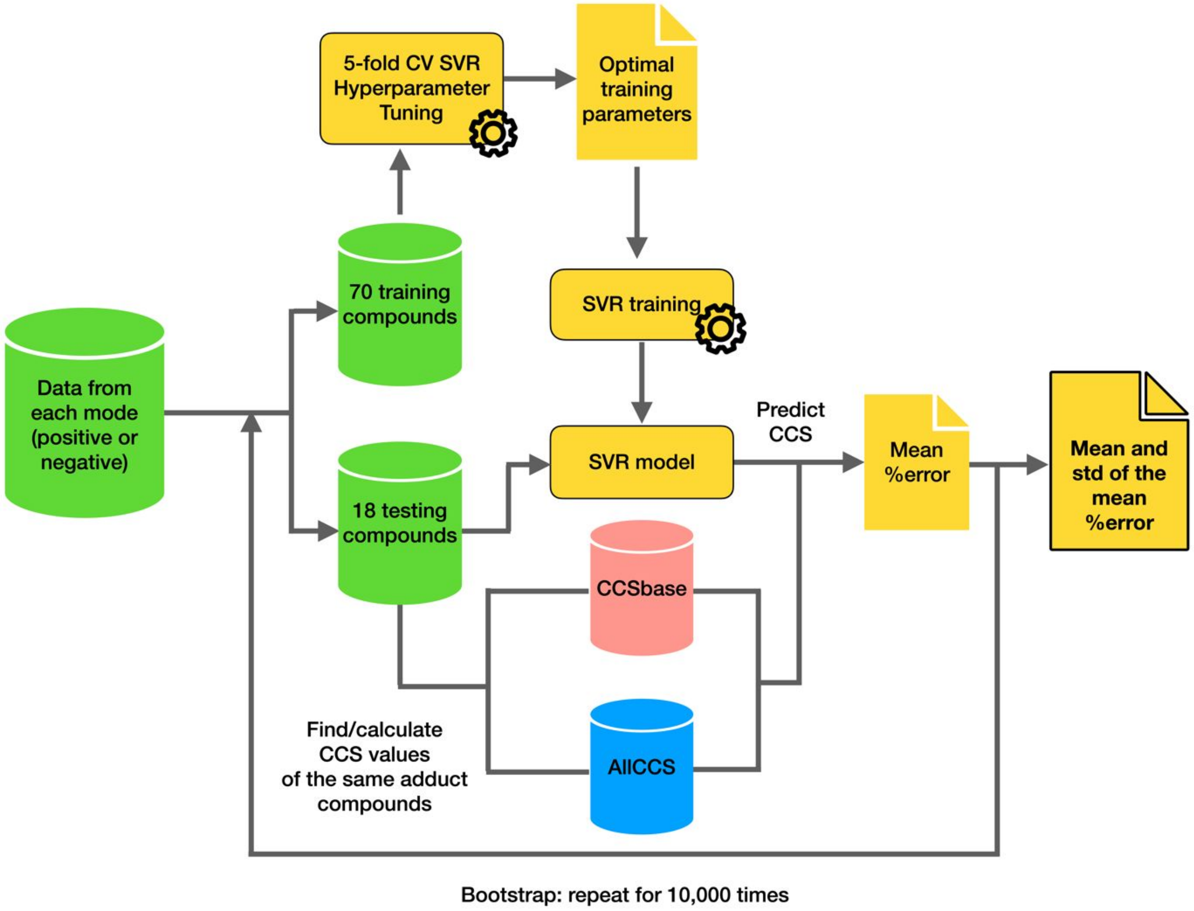

Supplementary Figure 8: Flowchart of SVR model building and the performance comparisons to existing databases and software. The data was subjected to a series of data partitioning in model evaluation process. The average percent errors were calculated and compared to indicate prediction performance of (1) our approach, (2) AllCCS, (3) CCSbase, and (4) IMoS.

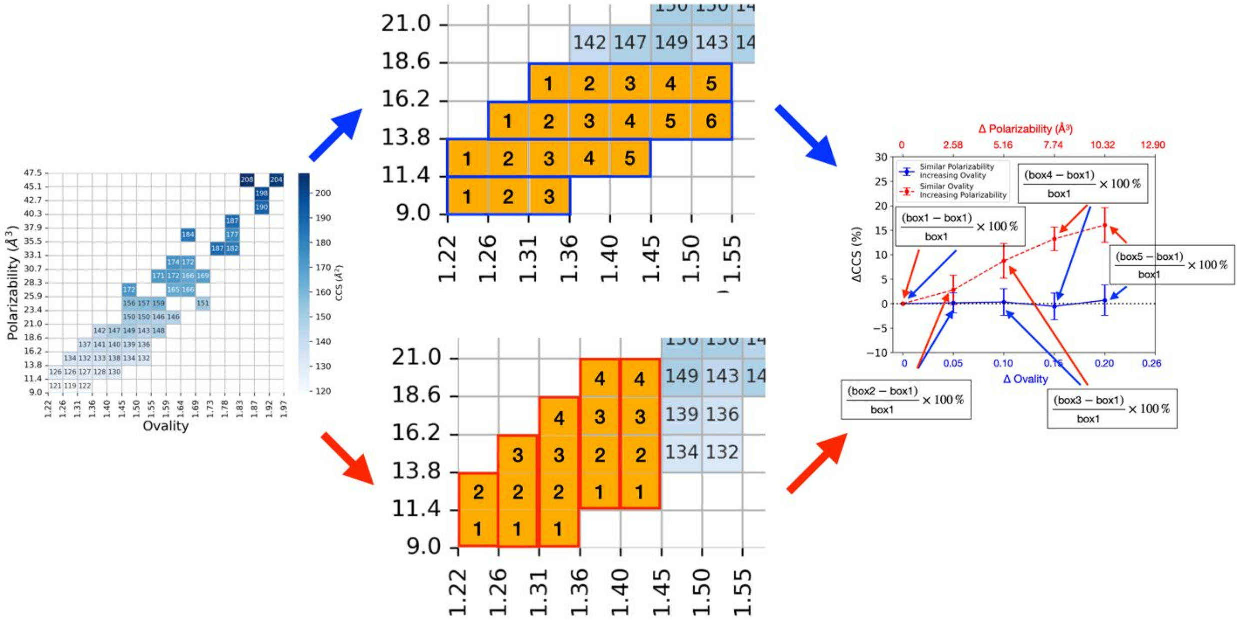

Supplementary Figure 9: Demonstration of the heat map analysis. The heat map of the CCS values between the polarizabilities and ovalities are used for this demonstration. The values on the heat map were compared horizontally (i.e., increasing ovality, constant polarizability) (upper, middle) and vertically (i.e., increasing polarizability, constant ovality) (lower, middle).

Table S1: Calculated molecular properties and measured CCS values of the adduct ions from our TWIMS experiment**.**

Name	HMDB ID	Adduct	MW	*m/z*	Our TWIMS CCS (Å^2)	VdW volume (Å^3)	VdW surface area (Å^2)	Gyration radius (Å)	Polarizability (Å^3)	Ovality
2-Aminobenzoic acid	HMDB01123	$[M+H]^+$	137.14	138.06	132.38	123.60	156.90	2.16	13.72	1.31
Adenine	HMDB00034	$[M+H]^+$	135.13	136.06	125.13	113.20	145.50	2.03	12.82	1.29
Adenosine	HMDB00050	$[M+H]^+$	267.24	268.11	155.89	222.00	276.00	3.32	25.61	1.56
AMP	HMDB00045	$[M+H]^+$	347.22	348.07	167.89	265.80	330.30	3.63	30.27	1.65
Arachidonic acid	HMDB01043	$[M+H]^+$	304.47	305.24	178.98	334.30	426.20	4.06	37.42	1.83
Ascorbic acid	HMDB00044	$[M+H]^+$	176.12	177.04	134.39	142.20	182.90	2.32	15.16	1.39
a-tocopherol	HMDB01893	$[M+H]^+$	430.71	431.39	216.53	476.40	597.30	7.03	56.75	2.02
Biotin	HMDB00030	$[M+H]^+$	244.31	245.10	148.62	213.70	264.60	3.20	25.35	1.53
Carnosine	HMDB00033	$[M+H]^+$	226.23	227.11	151.65	200.70	251.40	2.77	22.68	1.52
cGMP	HMDB01314	$[M+H]^+$	345.21	346.06	171.22	253.10	309.10	3.51	29.64	1.60
Creatine	HMDB00064	$[M+H]^+$	131.13	132.08	122.23	123.10	164.60	2.05	12.62	1.38
Creatinine	HMDB00562	$[M+H]^+$	113.12	114.07	120.17	102.30	138.70	1.92	11.19	1.31
Dopamine	HMDB00073	$[M+H]^+$	153.18	154.09	139.55	149.10	195.30	2.70	16.56	1.44
Eicosapentaenoic acid	HMDB01999	$[M+H]^+$	302.45	303.23	177.23	334.90	432.10	6.72	36.04	1.85
FAD	HMDB01248	$[M+H]^+$	785.55	786.16	250.07	605.90	717.00	5.36	71.54	2.07
Folic acid	HMDB00121	$[M+H]^+$	441.40	442.15	196.77	364.90	449.50	5.97	44.15	1.82
Glutathione	HMDB00125	$[M+H]^+$	307.32	308.09	166.01	260.70	330.90	4.45	29.55	1.68
Guanine	HMDB00132	$[M+H]^+$	151.13	152.06	128.49	121.00	157.10	2.20	13.85	1.33
Hydroxyproline	HMDB00725	$[M+H]^+$	131.13	132.07	125.85	119.10	156.80	2.12	12.67	1.34
Inosine	HMDB00195	$[M+H]^+$	268.23	269.09	155.86	215.80	264.90	3.14	24.94	1.52
L-Arginine	HMDB00517	$[M+H]^+$	174.20	175.12	136.63	165.50	221.90	3.17	18.34	1.52
L-Cystathionine	HMDB00099	$[M+H]^+$	222.26	223.08	144.00	193.30	252.10	3.51	22.52	1.56
L-Cystine	HMDB00192	$[M+H]^+$	240.30	241.03	146.43	196.60	249.80	2.88	22.95	1.53
L-Histidine	HMDB00177	$[M+H]^+$	155.15	156.08	131.08	138.40	179.60	2.59	15.12	1.39
L-Isoleucine	HMDB00172	$[M+H]^+$	131.17	132.10	131.15	135.60	179.30	2.09	14.57	1.40

L-Kynurenine	HMDB00684	$[M+H]^+$	208.21	209.09	146.07	185.00	227.90	3.11	20.90	1.45
L-Leucine	HMDB00687	$[M+H]^+$	131.17	132.10	132.88	136.40	180.30	2.24	14.63	1.41
L-Lysine	HMDB00182	$[M+H]^+$	146.19	147.11	131.69	150.00	208.50	2.99	16.32	1.53
L-Phenylalanine	HMDB00159	$[M+H]^+$	165.19	166.09	137.14	158.20	201.20	2.75	17.49	1.42
L-Proline	HMDB00162	$[M+H]^+$	115.13	116.07	123.70	108.60	144.70	1.95	11.79	1.31
L-Tryptophan	HMDB00929	$[M+H]^+$	204.23	205.10	147.76	183.70	227.80	3.01	21.47	1.46
L-Tyrosine	HMDB00158	$[M+H]^+$	181.19	182.08	141.09	163.70	207.40	3.01	18.51	1.43
L-Valine	HMDB00883	$[M+H]^+$	117.15	118.09	123.49	119.40	158.00	2.00	12.58	1.35
m-Cresol	HMDB02048	$[M+H]^+$	108.14	109.07	126.19	110.50	145.20	2.00	12.33	1.30
Methyl nonanoate	HMDB31264	$[M+H]^+$	172.26	173.16	148.93	196.40	265.90	3.84	21.93	1.63
Niacinamide	HMDB01406	$[M+H]^+$	122.12	123.06	123.01	112.10	146.80	2.13	12.15	1.31
Nicotinic acid	HMDB01488	$[M+H]^+$	123.11	124.04	122.92	108.50	138.60	2.10	11.51	1.26
Oxidized glutathione	HMDB03337	$[M+H]^+$	612.63	613.16	226.92	506.60	620.00	5.46	58.24	2.02
Pantothenic acid	HMDB00210	$[M+H]^+$	219.24	220.12	145.63	207.20	267.10	3.26	22.29	1.58
Phenylacetylglycine	HMDB00821	$[M+H]^+$	193.20	194.08	142.08	177.00	225.30	3.37	19.55	1.48
Pyroglutamic acid	HMDB00267	$[M+H]^+$	129.11	130.05	126.02	112.70	148.80	2.14	11.89	1.32
Riboflavin	HMDB00244	$[M+H]^+$	376.36	377.14	182.66	319.30	386.40	3.79	37.91	1.71
Spermidine	HMDB01257	$[M+H]^+$	145.25	146.17	135.15	169.50	231.70	3.36	18.92	1.56
Spermine	HMDB01256	$[M+H]^+$	202.34	203.22	150.86	232.50	316.40	4.74	26.74	1.73
Succinyl-CoA	HMDB01022	$[M+H]^+$	867.61	868.13	260.23	670.30	787.60	5.57	76.67	2.13
Taurine	HMDB00251	$[M+H]^+$	125.15	126.02	126.38	102.20	143.80	1.93	11.17	1.36
Thymine	HMDB00262	$[M+H]^+$	126.11	127.05	120.81	109.30	145.40	2.05	11.84	1.32
Uracil	HMDB00300	$[M+H]^+$	112.09	113.04	120.27	92.80	124.30	1.86	9.77	1.25
Uric acid	HMDB00289	$[M+H]^+$	168.11	169.04	133.36	123.10	160.20	2.41	14.04	1.34
Uridine	HMDB00296	$[M+H]^+$	244.20	245.08	146.30	196.70	240.80	2.89	22.18	1.47
Vitamin D3	HMDB00876	$[M+H]^+$	384.64	385.35	214.88	424.20	516.90	4.44	49.94	1.89
Xanthurenic acid	HMDB00881	$[M+H]^+$	205.17	206.05	136.81	165.20	200.50	2.76	19.24	1.38
3-Hydroxyphenylacetic acid	HMDB00440	$[M+Na]^+$	152.15	175.04	139.98	177.90	215.20	2.93	16.56	1.41
3-Hydroxyphenyllacetate	HMDB29232	$[M+Na]^+$	182.17	205.05	141.60	199.90	239.90	3.08	16.28	1.45

3-Methyladipic acid	HMDB00555	$[M+Na]^+$	160.17	183.06	136.22	190.00	232.50	2.59	17.70	1.45
3-Methylglutaconic acid	HMDB00522	$[M+Na]^+$	144.13	167.03	132.11	169.80	209.10	2.94	15.09	1.41
4-Hydroxyphenyl acetate	HMDB60390	$[M+Na]^+$	152.15	175.04	138.25	179.40	216.80	2.92	16.60	1.41
Adenine	HMDB00034	$[M+Na]^+$	135.13	158.04	134.02	150.10	177.80	2.26	14.30	1.30
Allantoin	HMDB00462	$[M+Na]^+$	158.12	181.03	136.32	160.80	193.80	2.54	14.79	1.36
Arachidic acid	HMDB02212	$[M+Na]^+$	312.53	335.29	198.31	399.10	513.90	7.66	44.83	1.96
Azelaic acid	HMDB00784	$[M+Na]^+$	188.22	211.09	145.99	222.50	271.50	3.01	22.40	1.53
Behenic acid	HMDB00944	$[M+Na]^+$	340.58	363.32	204.26	435.40	559.80	7.27	49.15	2.02
Betaine	HMDB00043	$[M+Na]^+$	118.15	140.07	132.22	158.40	194.60	2.33	14.07	1.37
Capric acid	HMDB00511	$[M+Na]^+$	172.26	195.14	143.53	229.60	293.50	4.27	23.53	1.62
Carnosine	HMDB00033	$[M+Na]^+$	226.23	249.10	152.28	238.80	291.30	3.18	24.20	1.56
cGMP	HMDB01314	$[M+Na]^+$	345.21	368.04	178.55	294.40	352.70	3.84	31.09	1.65
cis-Aconitic	HMDB00072	$[M+Na]^+$	174.11	197.01	139.19	176.70	212.60	2.63	15.89	1.40
Creatinine	HMDB00562	$[M+Na]^+$	113.12	136.05	129.07	144.20	182.30	2.50	12.80	1.37
D-Arabinose	HMDB29942	$[M+Na]^+$	150.13	173.04	131.78	170.50	206.00	2.57	15.22	1.39
D-Arabitol	HMDB00568	$[M+Na]^+$	152.15	175.06	133.34	174.50	213.80	2.59	16.30	1.42
D-Fructose	HMDB00660	$[M+Na]^+$	180.16	203.05	140.11	184.90	216.30	2.34	18.23	1.38
D-Galactose	HMDB00143	$[M+Na]^+$	180.16	203.05	138.53	190.70	221.10	2.51	17.92	1.38
D-Glucose	HMDB00122	$[M+Na]^+$	180.16	203.05	143.24	188.90	225.20	2.55	17.95	1.41
Diethyl methylsuccinate	HMDB59814	$[M+Na]^+$	188.22	211.09	151.23	220.10	269.00	2.99	21.94	1.53
D-Lactose	HMDB00186	$[M+Na]^+$	342.30	365.11	171.29	321.00	371.40	3.42	33.16	1.64
D-Maltose	HMDB00163	$[M+Na]^+$	342.30	365.10	175.45	317.90	373.30	3.51	33.00	1.66
D-Mannose	HMDB00169	$[M+Na]^+$	180.16	203.05	140.11	187.90	220.40	2.42	17.93	1.39
D-Ribose	HMDB00283	$[M+Na]^+$	150.13	173.04	131.78	167.50	205.10	2.33	14.58	1.40
D-Tagalose	HMDB03418	$[M+Na]^+$	180.16	203.05	136.93	185.90	218.30	2.43	17.88	1.39
D-Xylitol	HMDB02917	$[M+Na]^+$	152.15	175.06	133.34	174.10	213.10	2.55	16.18	1.41
D-Xylose	HMDB00098	$[M+Na]^+$	150.13	173.04	131.78	166.10	200.30	2.40	15.21	1.37
Erythritol	HMDB02994	$[M+Na]^+$	122.12	145.05	128.38	150.70	184.50	2.24	13.39	1.35
Folic acid	HMDB00121	$[M+Na]^+$	441.40	464.13	201.44	404.80	479.40	6.28	45.53	1.81

Fructose 6-phospahte	HMDB00124	$[M+Na]^+$	260.14	283.02	151.18	234.60	279.70	3.07	22.46	1.52
Galactitol	HMDB00107	$[M+Na]^+$	182.17	205.07	140.03	199.80	240.80	2.98	18.85	1.46
Heptanoic acid	HMDB00666	$[M+Na]^+$	130.18	153.09	134.35	181.40	230.20	3.27	17.35	1.49
Hippuric acid	HMDB00714	$[M+Na]^+$	179.17	202.05	146.37	195.70	229.20	2.97	19.31	1.41
Inosine	HMDB00195	$[M+Na]^+$	268.23	291.07	166.42	252.70	293.50	3.33	26.08	1.52
Linoleic acid	HMDB00673	$[M+Na]^+$	280.45	303.23	174.95	354.80	450.10	6.08	37.58	1.86
L-Kynurenine	HMDB00684	$[M+Na]^+$	208.21	231.07	149.75	226.30	264.80	3.37	22.65	1.47
L-Methionine	HMDB00696	$[M+Na]^+$	149.21	172.04	135.16	177.20	216.40	2.55	17.41	1.42
L-Proline	HMDB00162	$[M+Na]^+$	115.13	138.05	130.65	147.30	177.80	2.10	13.42	1.32
L-Tyrosine	HMDB00158	$[M+Na]^+$	181.19	204.06	143.20	202.70	245.50	3.41	20.29	1.47
Maltitol	HMDB02928	$[M+Na]^+$	344.31	367.12	174.13	327.00	378.90	3.48	33.42	1.65
Mannitol	HMDB00765	$[M+Na]^+$	182.17	205.07	140.03	195.20	229.60	2.62	18.81	1.41
myo-Inositol	HMDB00211	$[M+Na]^+$	180.16	203.05	146.00	184.60	216.30	2.40	17.94	1.38
Nicotinic acid	HMDB01488	$[M+Na]^+$	123.11	146.02	128.31	147.10	174.60	2.51	13.22	1.30
Nonadecanoic acid	HMDB00772	$[M+Na]^+$	298.50	321.28	190.13	390.80	500.50	6.78	42.88	1.94
Oxoadipic acid	HMDB00225	$[M+Na]^+$	160.12	183.03	136.98	178.10	222.00	3.26	16.09	1.45
Palmitic acid	HMDB00220	$[M+Na]^+$	256.42	279.23	179.96	331.70	428.40	5.66	36.27	1.85
Pectin	HMDB03402	$[M+Na]^+$	194.14	217.05	145.43	194.50	234.20	2.78	18.31	1.44
Ribitol	HMDB00508	$[M+Na]^+$	152.15	175.06	133.34	177.10	219.10	2.71	16.09	1.44
Sorbitol	HMDB00247	$[M+Na]^+$	182.17	205.07	140.03	200.90	241.40	2.75	18.93	1.46
Stearic acid	HMDB00827	$[M+Na]^+$	284.48	307.26	186.65	370.30	464.70	6.60	40.55	1.86
Sucrose	HMDB00258	$[M+Na]^+$	342.30	365.10	171.56	323.80	382.00	3.51	32.85	1.68
Sumiki's acid	HMDB02432	$[M+Na]^+$	142.11	165.02	129.97	158.90	195.40	2.82	14.86	1.38
trans-Aconitic acid	HMDB00958	$[M+Na]^+$	174.11	197.00	138.79	174.80	206.00	2.75	16.03	1.36
trans-Ferulic acid	HMDB00954	$[M+Na]^+$	194.18	217.05	161.96	211.00	252.00	3.19	21.43	1.47
Tropic acid	HMDB62590	$[M+Na]^+$	166.17	189.05	140.74	192.40	230.00	2.76	18.54	1.43
2-Aminobenzoic acid	HMDB01123	$[M-H]^-$	137.14	136.04	126.13	118.40	147.90	2.15	12.85	1.27
2-Aminobutyric acid	HMDB00452	$[M-H]^-$	103.12	102.06	121.74	97.70	134.60	1.91	10.00	1.31
2-Ethylhydracrylic acid	HMDB00396	$[M-H]^-$	118.13	117.05	123.21	111.80	149.60	1.89	11.50	1.33
2-Hydroxybutyric acid	HMDB00008	$[M-H]^-$	104.11	103.04	119.98	95.10	130.90	1.89	9.56	1.30

3-Hydroxyanthranilic acid	HMDB01476	[M-H]-	153.14	152.03	126.47	126.80	158.40	2.29	13.73	1.30
3-Hydroxybutyric acid	HMDB00011	[M-H]-	104.10	103.04	107.41	96.30	133.60	1.96	9.55	1.32
3-Hydroxyphenyllacetate	HMDB29232	[M-H]-	182.17	181.05	140.26	158.30	198.40	2.70	16.85	1.40
3-Methyladipic acid	HMDB00555	[M-H]-	160.17	159.07	131.84	185.70	232.20	2.95	15.55	1.48
4-Hydroxyphenyl acetate	HMDB60390	[M-H]-	152.15	151.04	129.47	133.00	170.90	2.62	14.42	1.36
Adenine	HMDB00034	[M-H]-	135.13	134.05	126.29	108.40	138.30	1.99	12.03	1.26
Adenosine	HMDB00050	[M-H]-	267.24	266.09	155.95	212.40	256.60	3.22	24.77	1.49
Allantoin	HMDB00462	[M-H]-	158.12	157.04	126.16	120.40	158.90	2.36	12.52	1.35
Arachidonic acid	HMDB01043	[M-H]-	304.47	303.23	186.98	335.00	422.60	3.63	36.70	1.81
Arginosuccinic acid	HMDB00052	[M-H]-	290.27	289.11	164.65	246.70	312.40	4.26	27.02	1.64
Ascorbic acid	HMDB00044	[M-H]-	176.12	175.02	122.95	137.50	174.60	2.41	14.58	1.36
α-Tocopherol	HMDB01893	[M-H]-	430.71	429.37	212.59	470.50	582.60	7.07	55.97	1.99
ATP	HMDB00538	[M-H]-	507.18	505.99	190.77	342.10	415.80	4.39	37.85	1.76
Azelaic acid	HMDB00784	[M-H]-	188.22	187.10	139.96	181.70	241.00	3.62	20.03	1.55
Capric acid	HMDB00511	[M-H]-	172.26	171.14	151.28	186.40	251.30	3.89	21.14	1.59
Caprylic acid	HMDB00482	[M-H]-	144.21	143.11	141.36	152.70	207.30	2.98	16.95	1.50
Carnosine	HMDB00033	[M-H]-	226.23	225.10	154.88	198.20	251.10	3.11	21.84	1.53
cGMP	HMDB01314	[M-H]-	345.21	344.04	172.34	247.40	288.80	3.27	28.84	1.52
Creatinine	HMDB00562	[M-H]-	113.12	112.05	120.56	98.90	133.30	1.90	10.45	1.29
D-Arabitol	HMDB00568	[M-H]-	152.15	151.06	126.54	135.50	180.70	2.40	14.28	1.42
D-Lactose	HMDB00186	[M-H]-	342.30	341.11	172.39	278.40	335.60	3.25	30.95	1.63
D-Mannose	HMDB00169	[M-H]-	180.16	179.05	132.14	145.70	177.60	2.13	15.96	1.33
Dopamine	HMDB00073	[M-H]-	153.18	152.07	130.85	142.90	187.10	2.69	15.84	1.42
D-Ribose	HMDB00283	[M-H]-	150.13	149.04	121.34	121.80	153.50	1.99	13.12	1.29
D-Xylitol	HMDB02917	[M-H]-	152.15	151.06	128.01	134.50	175.20	2.40	13.99	1.38
Eicosapentaenoic acid	HMDB01999	[M-H]-	302.45	301.22	190.11	331.10	420.40	6.85	35.37	1.82
Epinephrine	HMDB00068	[M-H]-	183.20	182.08	140.21	168.30	214.20	2.83	18.56	1.45
FAD	HMDB01248	[M-H]-	785.55	784.15	243.71	595.60	688.50	5.61	70.46	2.01

Folic acid	HMDB00121	$[M-H]^-$	441.40	440.13	187.54	360.50	434.00	6.00	43.19	1.77
Fructose 1,6-bisphosphate	HMDB01058	$[M-H]^-$	340.12	339.00	159.01	237.20	296.50	3.19	25.44	1.60
Fructose 6-phosphate	HMDB00124	$[M-H]^-$	260.14	259.02	146.38	195.20	246.00	2.88	20.56	1.51
Gentisic acid	HMDB00152	$[M-H]^-$	154.12	153.02	126.41	121.70	151.20	2.33	13.33	1.27
Glutathione	HMDB00125	$[M-H]^-$	307.32	306.08	166.50	256.90	328.80	4.27	28.86	1.68
Guanine	HMDB00132	$[M-H]^-$	151.13	150.04	125.12	115.70	148.40	2.17	12.96	1.29
Hippuric acid	HMDB00714	$[M-H]^-$	179.17	178.05	139.07	158.50	199.50	2.99	17.14	1.41
Hydroxyproline	HMDB00725	$[M-H]^-$	131.13	130.05	126.62	114.70	150.10	2.07	11.87	1.32
Indoleacetic acid	HMDB00197	$[M-H]^-$	175.18	174.06	141.98	154.10	192.80	2.57	17.36	1.39
Inosine	HMDB00195	$[M-H]^-$	268.23	267.07	159.47	210.20	252.40	3.15	24.07	1.48
Isobutyric acid	HMDB01873	$[M-H]^-$	88.11	87.04	118.84	87.40	123.40	1.66	8.70	1.30
Isovaleric acid	HMDB00718	$[M-H]^-$	102.13	101.06	123.48	103.90	143.50	1.98	10.59	1.34
L-Alanine	HMDB00161	$[M-H]^-$	89.09	88.04	117.06	82.00	115.60	1.64	8.13	1.27
L-Arginine	HMDB00517	$[M-H]^-$	174.20	173.10	136.62	160.00	212.90	3.19	17.58	1.49
L-Asparagine	HMDB00168	$[M-H]^-$	132.12	131.04	123.48	110.90	147.50	2.28	11.38	1.32
L-Aspartic acid	HMDB00191	$[M-H]^-$	133.10	132.03	120.30	107.70	143.40	2.06	10.99	1.31
L-Cystine	HMDB00192	$[M-H]^-$	240.30	239.02	143.16	191.50	241.40	2.93	22.11	1.50
L-Histidine	HMDB00177	$[M-H]^-$	155.15	154.06	129.28	132.50	174.00	2.60	14.37	1.38
L-Leucine	HMDB00687	$[M-H]^-$	131.17	130.09	131.10	132.50	179.20	2.20	13.80	1.43
L-Lysine	HMDB00182	$[M-H]^-$	146.19	145.10	135.63	146.30	198.00	2.99	15.56	1.47
L-Phenylalanine	HMDB00159	$[M-H]^-$	165.19	164.07	138.51	152.90	191.40	2.50	16.74	1.38
L-Proline	HMDB00162	$[M-H]^-$	115.13	114.06	116.46	107.00	143.70	1.97	11.01	1.32
L-Serine	HMDB00187	$[M-H]^-$	105.09	104.03	118.21	90.00	124.40	1.88	9.03	1.28
L-Threonine	HMDB00167	$[M-H]^-$	119.12	118.05	121.54	106.80	142.10	1.91	10.75	1.31
L-Tryptophan	HMDB00929	$[M-H]^-$	204.23	203.08	148.33	182.10	226.70	3.02	20.72	1.46
L-Tyrosine	HMDB00158	$[M-H]^-$	181.19	180.07	141.65	161.50	204.30	3.03	17.74	1.42
L-Valine	HMDB00883	$[M-H]^-$	117.15	116.07	126.43	115.70	156.40	1.98	11.83	1.36
Malic acid	HMDB00156	$[M-H]^-$	134.09	133.01	118.53	103.80	137.20	2.12	10.49	1.28
m-Cresol	HMDB02048	$[M-H]^-$	108.14	107.05	122.73	104.10	134.90	1.96	11.52	1.26

Methyl heptanoate	HMDB31478	$[M-H]^-$	144.21	143.11	137.20	153.60	206.90	3.19	17.12	1.49
Methyl nonanoate	HMDB31264	$[M-H]^-$	172.26	171.14	144.80	188.60	255.60	3.64	21.31	1.61
Myo-Inositol	HMDB00211	$[M-H]^-$	180.16	179.05	133.54	148.40	181.80	2.16	15.79	1.34
Myristic acid	HMDB00806	$[M-H]^-$	228.37	227.20	168.91	257.30	341.20	5.51	29.63	1.74
Nicotinic acid	HMDB01488	$[M-H]^-$	123.11	122.02	121.16	102.40	132.20	2.08	10.86	1.25
Oxidized glutathione	HMDB03337	$[M-H]^-$	612.63	611.14	216.04	501.50	619.60	5.13	57.56	2.03
Oxoadipic acid	HMDB00225	$[M-H]^-$	160.12	159.03	128.96	134.60	183.00	3.09	13.74	1.44
Pantothenic acid	HMDB00210	$[M-H]^-$	219.24	218.10	146.44	200.70	258.20	3.40	21.56	1.56
Phenylacetylglycine	HMDB00821	$[M-H]^-$	193.20	192.07	146.28	172.60	221.50	3.26	18.70	1.48
Pyroglutamic acid	HMDB00267	$[M-H]^-$	129.11	128.03	123.74	107.80	143.20	2.12	11.15	1.31
Quinolinic acid	HMDB00232	$[M-H]^-$	167.12	166.02	128.56	129.70	164.20	2.31	13.92	1.33
Ribitol	HMDB00508	$[M-H]^-$	152.15	151.06	126.54	130.80	170.10	2.23	14.06	1.37
Riboflavin	HMDB00244	$[M-H]^-$	376.36	375.13	184.43	316.80	375.00	3.71	37.20	1.67
Sorbitol	HMDB00247	$[M-H]^-$	182.17	181.07	133.44	158.10	201.70	2.50	16.83	1.43
Succinic acid	HMDB00254	$[M-H]^-$	118.09	117.02	118.44	98.20	136.30	2.32	9.76	1.32
Sucrose	HMDB00258	$[M-H]^-$	342.30	341.11	169.11	278.40	335.80	3.27	30.87	1.63
Sumiki's acid	HMDB02432	$[M-H]^-$	142.11	141.02	128.72	115.90	154.80	2.35	12.55	1.35
Taurine	HMDB00251	$[M-H]^-$	125.15	124.01	119.39	96.60	135.80	1.91	10.49	1.33
trans-Aconitic	HMDB00958	$[M-H]^-$	174.11	173.00	118.87	133.50	166.70	2.41	13.61	1.32
trans-Ferulic acid	HMDB00954	$[M-H]^-$	194.18	193.05	143.64	170.80	212.70	2.84	18.84	1.43
Uracil	HMDB00300	$[M-H]^-$	112.09	111.02	115.77	86.80	115.40	1.84	8.99	1.22
Uric acid	HMDB00289	$[M-H]^-$	168.11	167.02	125.59	119.50	154.s00	2.40	13.24	1.31
Uridine	HMDB00296	$[M-H]^-$	244.20	243.06	153.03	193.70	239.40	3.10	21.34	1.48
Valeric acid	HMDB00892	$[M-H]^-$	102.13	101.06	123.48	104.10	144.10	2.30	10.80	1.35
Vanillylmandelic acid	HMDB00291	$[M-H]^-$	198.17	197.05	144.75	167.00	206.80	2.79	18.00	1.41
Vitamin D3	HMDB00876	$[M-H]^-$	384.64	383.33	201.16	419.30	513.40	4.44	49.27	1.89
Xanthurenic acid	HMDB00881	$[M-H]^-$	205.17	204.03	136.52	163.90	197.50	2.76	18.58	1.36

Table S2: Calculated molecular properties and CCS values only for the adducts found in both our experiment and CCSbase's DTIMS experimental database. The HMDB ID of the compounds listed here can be found in Table S1.

Name	Adduct	m/z	CCSbase DTIMS CCS (Å²)	Our TWIMS CCS (Å²)	VdW volume (Å³)	VdW surface area (Å²)	Gyration radius (Å)	Polarizability (Å³)	Ovality
Adenine	$[M+H]^+$	136.06	125.97	125.13	113.20	145.50	2.03	12.82	1.29
Adenosine	$[M+H]^+$	268.11	157.42	155.89	222.00	276.00	3.32	25.61	1.56
Ascorbic acid	$[M+H]^+$	177.04	138.60	134.39	142.20	182.90	2.32	15.16	1.39
Carnosine	$[M+H]^+$	227.11	151.33	151.65	200.70	251.40	2.77	22.68	1.52
cGMP	$[M+H]^+$	346.06	172.80	171.22	253.10	309.10	3.51	29.64	1.60
Creatinine	$[M+H]^+$	114.07	123.60	120.17	102.30	138.70	1.92	11.19	1.31
Dopamine	$[M+H]^+$	154.09	133.77	139.55	149.10	195.30	2.70	16.56	1.44
FAD	$[M+H]^+$	786.16	249.00	250.07	605.90	717.00	5.36	71.54	2.07
Glutathione	$[M+H]^+$	308.09	167.40	166.01	260.70	330.90	4.45	29.55	1.68
Guanine	$[M+H]^+$	152.06	131.72	128.49	121.00	157.10	2.20	13.85	1.33
Inosine	$[M+H]^+$	269.09	159.25	155.86	215.80	264.90	3.14	24.94	1.52
Arginine	$[M+H]^+$	175.12	136.61	136.63	165.50	221.90	3.17	18.34	1.52
Cystine	$[M+H]^+$	241.03	149.63	146.43	196.60	249.80	2.88	22.95	1.53
Histidine	$[M+H]^+$	156.08	132.17	131.08	138.40	179.60	2.59	15.12	1.39
Isoleucine	$[M+H]^+$	132.10	134.59	131.15	135.60	179.30	2.09	14.57	1.40
Kynurenine	$[M+H]^+$	209.09	149.77	146.07	185.00	227.90	3.11	20.90	1.45
Leucine	$[M+H]^+$	132.10	135.17	132.88	136.40	180.30	2.24	14.63	1.41
Phenylalanine	$[M+H]^+$	166.09	140.59	137.14	158.20	201.20	2.75	17.49	1.42
Proline	$[M+H]^+$	116.07	125.58	123.70	108.60	144.70	1.95	11.79	1.31
Tryptophan	$[M+H]^+$	205.10	151.54	147.76	183.70	227.80	3.01	21.47	1.46
Tyrosine	$[M+H]^+$	182.08	145.98	141.09	163.70	207.40	3.01	18.51	1.43
Valine	$[M+H]^+$	118.09	127.59	123.49	119.40	158.00	2.00	12.58	1.35
Nicotinic acid	$[M+H]^+$	124.04	128.35	122.92	108.50	138.60	2.10	11.51	1.26

Pantothenic acid	$[M+H]^+$	220.12	150.17	145.63	207.20	267.10	3.26	22.29	1.58
Pyroglutamic acid	$[M+H]^+$	130.05	130.60	126.02	112.70	148.80	2.14	11.89	1.32
Spermine	$[M+H]^+$	203.22	148.40	150.86	232.50	316.40	4.74	26.74	1.73
Taurine	$[M+H]^+$	126.02	131.20	126.38	102.20	143.80	1.93	11.17	1.36
Thymine	$[M+H]^+$	127.05	123.38	120.81	109.30	145.40	2.05	11.84	1.32
Uracil	$[M+H]^+$	113.04	122.74	120.27	92.80	124.30	1.86	9.77	1.25
Uric acid	$[M+H]^+$	169.04	131.00	133.36	123.10	160.20	2.41	14.04	1.34
Xanthurenic acid	$[M+H]^+$	206.05	140.50	136.81	165.20	200.50	2.76	19.24	1.38
Adenine	$[M+Na]^+$	158.04	137.20	134.02	150.10	177.80	2.26	14.30	1.30
Allantoin	$[M+Na]^+$	181.03	143.91	136.32	160.80	193.80	2.54	14.79	1.36
Arachidic acid	$[M+Na]^+$	335.29	199.97	198.31	399.10	513.90	7.66	44.83	1.96
Betaine	$[M+Na]^+$	140.07	134.38	132.22	158.40	194.60	2.33	14.07	1.37
Carnosine	$[M+Na]^+$	249.10	154.70	152.28	238.80	291.30	3.18	24.20	1.56
Creatinine	$[M+Na]^+$	136.05	133.07	129.07	144.20	182.30	2.50	12.80	1.37
Arabinose	$[M+Na]^+$	173.04	139.91	131.78	170.50	206.00	2.57	15.22	1.39
Arabitol	$[M+Na]^+$	175.06	135.80	133.34	174.50	213.80	2.59	16.30	1.42
Fructose	$[M+Na]^+$	203.05	141.61	140.11	184.90	216.30	2.34	18.23	1.38
Galactose	$[M+Na]^+$	203.05	142.15	138.53	190.70	221.10	2.51	17.92	1.38
Glucose	$[M+Na]^+$	203.05	147.72	143.24	188.90	225.20	2.55	17.95	1.41
Lactose	$[M+Na]^+$	365.11	176.83	171.29	321.00	371.40	3.42	33.16	1.64
Maltose	$[M+Na]^+$	365.10	178.73	175.45	317.90	373.30	3.51	33.00	1.66
Mannose	$[M+Na]^+$	203.05	141.37	140.11	187.90	220.40	2.42	17.93	1.39
Xylitol	$[M+Na]^+$	175.06	137.16	133.34	174.10	213.10	2.55	16.18	1.41
Xylose	$[M+Na]^+$	173.04	134.75	131.78	166.10	200.30	2.40	15.28	1.37
Erythritol	$[M+Na]^+$	145.05	129.90	128.38	150.70	184.50	2.24	13.39	1.35
Hippuric acid	$[M+Na]^+$	202.05	154.06	146.37	195.70	229.20	2.97	19.31	1.41
Inosine	$[M+Na]^+$	291.07	172.29	166.42	252.70	293.50	3.33	26.08	1.52
Kynurenine	$[M+Na]^+$	231.07	152.95	149.75	226.30	264.80	3.37	22.65	1.47
Tyrosine	$[M+Na]^+$	204.06	143.78	143.20	202.70	245.50	3.41	20.29	1.47
Mannitol	$[M+Na]^+$	205.07	140.90	140.03	195.20	229.60	2.62	18.81	1.41

Myo-Inositol	[M+Na]+	203.05	148.20	146.00	184.60	216.30	2.40	17.94	1.38
Palmitic acid	[M+Na]+	279.23	190.11	179.96	331.70	428.40	5.66	36.27	1.85
Ribitol	[M+Na]+	175.06	136.25	133.34	177.10	219.10	2.71	16.09	1.44
Stearic acid	[M+Na]+	307.26	191.83	186.65	370.30	464.70	6.60	40.55	1.86
Sucrose	[M+Na]+	365.10	173.94	171.56	323.80	382.00	3.51	32.85	1.68
2-Hydroxybutyric acid	[M-H]-	103.04	124.67	119.98	95.10	130.90	1.89	9.56	1.30
3-Methyladipic acid	[M-H]-	159.07	129.01	131.84	185.70	232.20	2.95	15.55	1.48
Adenine	[M-H]-	134.05	120.57	126.29	108.40	138.30	1.99	12.03	1.26
Adenosine	[M-H]-	266.09	159.87	155.95	212.40	256.60	3.22	24.77	1.49
Allantoin	[M-H]-	157.04	126.95	126.16	120.40	158.90	2.36	12.52	1.35
Arachidonic acid	[M-H]-	303.23	181.60	186.98	335.00	422.60	3.63	36.70	1.81
Ascorbic acid	[M-H]-	175.02	130.67	122.95	137.50	174.60	2.41	14.58	1.36
Azelaic acid	[M-H]-	187.10	137.38	139.96	181.70	241.00	3.62	20.03	1.55
Capric acid	[M-H]-	171.14	146.87	151.28	186.40	251.30	3.89	21.14	1.59
Caprylic acid	[M-H]-	143.11	139.46	141.36	152.70	207.30	2.98	16.95	1.50
Carnosine	[M-H]-	225.10	152.71	154.88	198.20	251.10	3.11	21.84	1.53
Creatinine	[M-H]-	112.05	119.13	120.56	98.90	133.30	1.90	10.45	1.29
Arabitol	[M-H]-	151.06	124.90	126.54	135.50	180.70	2.40	14.28	1.42
Lactose	[M-H]-	341.11	173.57	172.39	278.40	335.60	3.25	30.95	1.63
Dopamine	[M-H]-	152.07	132.22	130.85	142.90	187.10	2.69	15.84	1.42
Xylitol	[M-H]-	151.06	125.04	128.01	134.50	175.20	2.40	13.99	1.38
Eicosapentaenoic acid	[M-H]-	301.22	178.53	190.11	331.10	420.40	6.85	35.37	1.82
FAD	[M-H]-	784.15	238.70	243.71	595.60	688.50	5.61	70.46	2.01
Fructose 6-phosphate	[M-H]-	259.02	144.10	146.38	195.20	246.00	2.88	20.56	1.51
Gentisic acid	[M-H]-	153.02	122.67	126.41	121.70	151.20	2.33	13.33	1.27
Glutathione	[M-H]-	306.08	164.24	166.50	256.90	328.80	4.27	28.86	1.68
Hippuric acid	[M-H]-	178.05	143.97	139.07	158.50	199.50	2.99	17.14	1.41
Inosine	[M-H]-	267.07	159.95	159.47	210.20	252.40	3.15	24.07	1.48

Asparagine	[M-H]-	131.04	124.51	123.48	110.90	147.50	2.28	11.38	1.32
Aspartic acid	[M-H]-	132.03	120.27	120.30	107.70	143.40	2.06	10.99	1.31
Cystine	[M-H]-	239.02	143.70	143.16	191.50	241.40	2.93	22.11	1.50
Histidine	[M-H]-	154.06	129.52	129.28	132.50	174.00	2.60	14.37	1.38
Leucine	[M-H]-	130.09	134.40	131.10	132.50	179.20	2.20	13.80	1.43
Lysine	[M-H]-	145.10	136.32	135.63	146.30	198.00	2.99	15.56	1.47
Phenylalanine	[M-H]-	164.07	142.40	138.51	152.90	191.40	2.50	16.74	1.38
Proline	[M-H]-	114.06	126.83	116.46	107.00	143.70	1.97	11.01	1.32
Serine	[M-H]-	104.03	136.66	118.21	90.00	124.40	1.88	9.03	1.28
Threonine	[M-H]-	118.05	130.78	121.54	106.80	142.10	1.91	10.75	1.31
Tryptophan	[M-H]-	203.08	148.72	148.33	182.10	226.70	3.02	20.72	1.46
Tyrosine	[M-H]-	180.07	145.88	141.65	161.50	204.30	3.03	17.74	1.42
Valine	[M-H]-	116.07	128.59	126.43	115.70	156.40	1.98	11.83	1.36
Myristic acid	[M-H]-	227.20	159.21	168.91	257.30	341.20	5.51	29.63	1.74
Nicotinic acid	[M-H]-	122.02	124.50	121.16	102.40	132.20	2.08	10.86	1.25
Pantothenic acid	[M-H]-	218.10	149.87	146.44	200.70	258.20	3.40	21.56	1.56
Phenylacetylglycine	[M-H]-	192.07	147.23	146.28	172.60	221.50	3.26	18.70	1.48
Quinolinic acid	[M-H]-	166.02	130.03	128.56	129.70	164.20	2.31	13.92	1.33
Ribitol	[M-H]-	151.06	125.80	126.54	130.80	170.10	2.23	14.06	1.37
Sorbitol	[M-H]-	181.07	130.52	133.44	158.10	201.70	2.50	16.83	1.43
Succinic acid	[M-H]-	117.02	119.84	118.44	98.20	136.30	2.32	9.76	1.32
Sucrose	[M-H]-	341.11	168.34	169.11	278.40	335.80	3.27	30.87	1.63
Taurine	[M-H]-	124.01	118.40	119.39	96.60	135.80	1.91	10.49	1.33
Uracil	[M-H]-	111.02	112.90	115.77	86.80	115.40	1.84	8.99	1.22
Uric acid	[M-H]-	167.02	127.01	125.59	119.50	154.00	2.40	13.24	1.31
Uridine	[M-H]-	243.06	152.42	153.03	193.70	239.40	3.10	21.34	1.48
Xanthurenic acid	[M-H]-	204.03	132.51	136.52	163.90	197.50	2.76	18.58	1.36

Table S3: Performance comparison between our models, AllCCS, and CCSbase in the positive and negative ion modes. The values listed in this table are the mean relative errors and their standard deviations from 10,000 bootstrapped runs. The polarizability of the analyte in the adduct form and neutral form are denoted by *P* and *P**, respectively. The *P+m/z* model outperforms the others in the positive mode.

**Predictor**	**MRE (ave ± std) (%)**	
	**Positive**	**Negative**
P + m/z	1.75 ± 0.35	2.10 ± 0.51
P* + m/z	1.97 ± 0.50	2.10 ± 0.48
**AllCCS**	2.72 ± 0.42	2.20 ± 0.47
**CCSbase**	2.04 ± 0.34	2.18 ± 0.47
